# Pyrrolizidine Alkaloids—Pros and Cons for Pharmaceutical and Medical Applications

**DOI:** 10.3390/ijms242316972

**Published:** 2023-11-30

**Authors:** Kavindi Jayawickreme, Dawid Świstak, Ewa Ozimek, Emilia Reszczyńska, Anna Rysiak, Anna Makuch-Kocka, Agnieszka Hanaka

**Affiliations:** 1 Student Scientific Club of Phytochemists, Institute of Biological Sciences, Faculty of Biology and Biotechnology, Maria Curie-Skłodowska University, Akademicka St. 19, 20-033 Lublin, Poland; 2 Department of Industrial and Environmental Microbiology, Institute of Biological Sciences, Faculty of Biology and Biotechnology, Maria Curie-Skłodowska University, Akademicka St. 19, 20-033 Lublin, Poland; 3 Department of Biochemistry and Molecular Biology, Medical University of Lublin, Chodźki St. 1, 20-093 Lublin, Poland; 4 Department of Plant Physiology and Biophysics, Institute of Biological Sciences, Faculty of Biology and Biotechnology, Maria Curie-Skłodowska University, Akademicka St. 19, 20-033 Lublin, Poland; 5 Department of Botany, Mycology, and Ecology, Institute of Biological Sciences, Faculty of Biology and Biotechnology, Maria Curie-Skłodowska University, Akademicka St. 19, 20-033 Lublin, Poland; 6 Department of Pharmacology, Medical University of Lublin, Radziwiłłowska St. 11, 20-080 Lublin, Poland

**Keywords:** pyrrolizidine alkaloid, hepatotoxicity, cytochrome P450, genotoxicity, food safety, *Boraginaceae*, *Streptomyces*, microbial alkaloids, anticancer activity, antimicrobial activity

## Abstract

Heterocyclic organic compounds named pyrrolizidine alkaloids (PAs) belong to a group of alkaloids and are synthesized by either plants or microorganisms. Therefore, they are naturally occurring secondary metabolites. They are found in species applied in the pharmaceutical and food industries, thus a thorough knowledge of their pharmacological properties and toxicology to humans is of great importance for their further safe employment. This review is original because it synthesizes knowledge of plant and microbial PAs, which is unusual in the scientific literature. We have focused on the *Boraginaceae* family, which is unique due to the exceptional richness and diversity of its PAs in plant species. We have also presented the microbial sources of PAs, both from fungi and bacteria. The structure and metabolism of PAs have been discussed. Our main aim was to summarize the effects of PAs on humans, including both negative, toxic ones, mainly concerning hepatotoxicity and carcinogenicity, as well as potentially positive ones for pharmacological and medical applications. We have collected the results of studies on the anticancer activity of PAs from plant and microbial sources (mainly *Streptomyces* strains) and on the antimicrobial activity of PAs on different strains of microorganisms (bacteria and fungi). Finally, we have suggested potential applications and future perspectives.

## 1. Introduction

Pyrrolizidine alkaloids (PAs) are synthesized by either plants or microorganisms and are referred to as secondary metabolites [1,2]. In flora, PAs are widely distributed with over 600 different compounds produced by more than 6000 angiosperm species, accounting for 3% of all species [1,3,4,5]. However, they occur mainly in certain families and, in turn, generally only in some tribes or genera. Pyrrolizidine alkaloids are prevalent in six families: *Apocynaceae, Ranunculaceae, Scrophulariaceae, Fabaceae, Asteraceae,* and *Boraginaceae*. The first four families mentioned above contain PAs in certain genera or species, while the *Asteraceae* family has diverse and widespread alkaloids. On the contrary, these compounds are present in every genus of the *Boraginaceae* family. Determined concentrations range widely, from trace amounts to 19% of dry weight, and they depend on several factors, such as the plant age and part, growth conditions, and procedures of extraction and analysis [1,6]. 

In contrast to the considerable understanding of plant PAs, there is limited knowledge regarding microbial PAs. Currently, approx. 40 bacterial PAs have been identified, with groups such as clazamycins, bohemamines, and jenamidines commonly synthesized by *Streptomyces* species [2,7]. A smaller number of PA molecules have been found in fungi, with a group of lolines serving as an example [8].

Pyrrolizidine alkaloids are believed to function as self-defense metabolites, protecting plants against herbivores (due to the confirmed inhibitory effect of specific PAs on acetylcholinesterase activity) [9,10], or defending bacteria against predators such as amoeba [11]. These compounds are notorious for their hepatotoxic and carcinogenic effects on mammals including cows, horses, and humans [12,13,14]. Nowadays, industries require a variety of compounds with different activity spectra. In pharmaceutical and medical research, natural remedies derived from plants and microorganisms are gaining interest in addition to synthetic preparations. The compounds belonging to PAs can be toxic and may have diverse side effects. On the other hand, they exhibit therapeutic effects, such as anticancer or antimicrobial activities. Therefore, the aim of this review is to summarize the effects of plant- and microorganism-derived PAs on humans, including both harmful, toxic ones, as well as potentially beneficial implications for pharmacology and medicine.

## 2. Chemical Structure and General Characteristics of PAs

**Figure 1:**
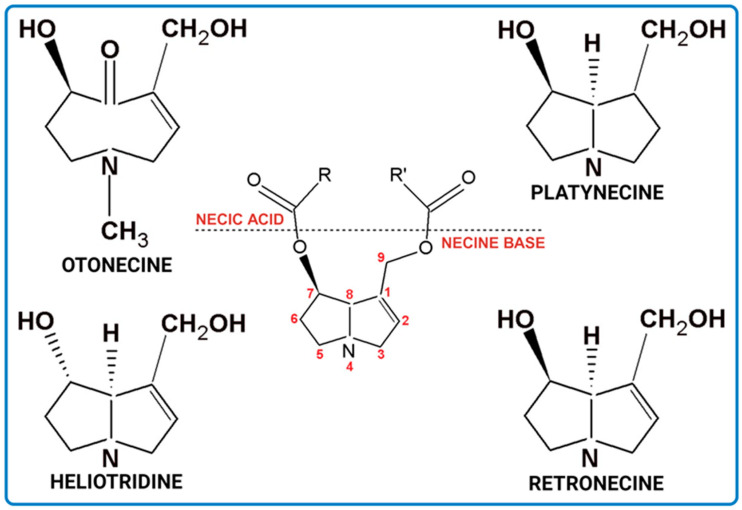
Structure of PA ester and types of necine bases (ACD/ChemSketch (Freewere); BioRender, 2023).

Pyrrolizidine alkaloids are present in two main chemical forms, which are tertiary amines (1,2-dehydropyrrolizidine alkaloids) and corresponding *N*-oxides [10,15]. They primarily exist as esters composed of a necine base and at least one necic acid [4,10] (Figure 1). Based on the structure of esters, four major groups of PAs are formed, i.e., 11- and 12-ring macrocyclic diesters, as well as open-chained mono- or diesters [1,16]. In addition, there may be occasional occurrences of other uncommon forms, such as 13-ring macrocyclic diesters or aromatic acids [4,10].

The necine base comprises of pyrrolizidine, an amino alcohol consisting of two five-membered rings connected by a single nitrogen atom [4,10]. Four standard types of necine bases are categorized as retronecine, heliotridine, otonecine, and platynecine (Figure 1). They differ in the ring cyclicity and saturation of the double bond, which is linked to their toxicity [1,10] (Table 1). Additionally, retronecine- and heliotridine-type PAs are enantiomers of one another at the C7 position, where they possess R and S stereochemistry, respectively [17].

The necic acid can be a mono-, dicarboxylic aliphatic acid, or a monocarboxylic aromatic acid [1,10]. The combination of necine bases and necic acids, as well as their linkage patterns, has resulted in the classification of PAs into eight groups [1] (Table 2).

*N*-oxides are significantly more predominant in plant species than tertiary bases. They are derived from the aforementioned bases but possess distinct properties [18]. *N*-oxides are highly water-soluble and considered less toxic than free forms (a pyrrolizidine base), making them metabolically safe [1,15]. 

**Figure 2:**
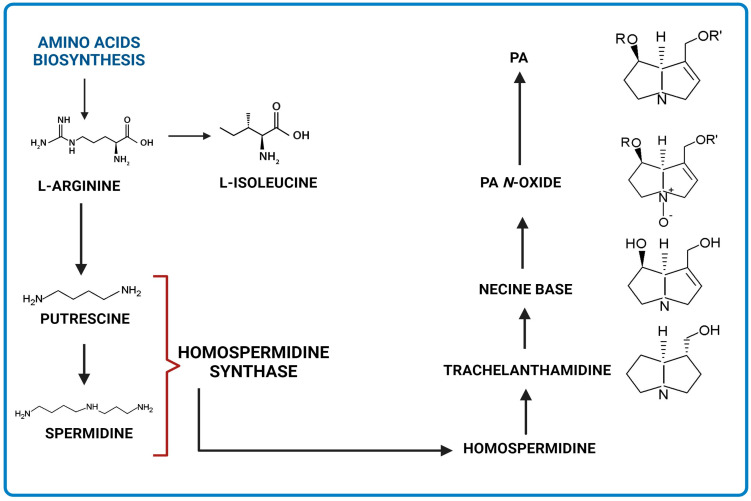
Simplified scheme of PAs biosynthesis (ACD/ChemSketch (Freewere); BioRender, 2023).

Biosynthesis of PAs is presented in Figure 2. The precursors are amino acid molecules. The key enzyme is homospermidine synthase, which links the primary and secondary metabolism. Due to its activity, homosperimidine is formed and subsequently incorporated into the necine base of PA [1,2] (Figure 2). 

**Figure 3:**
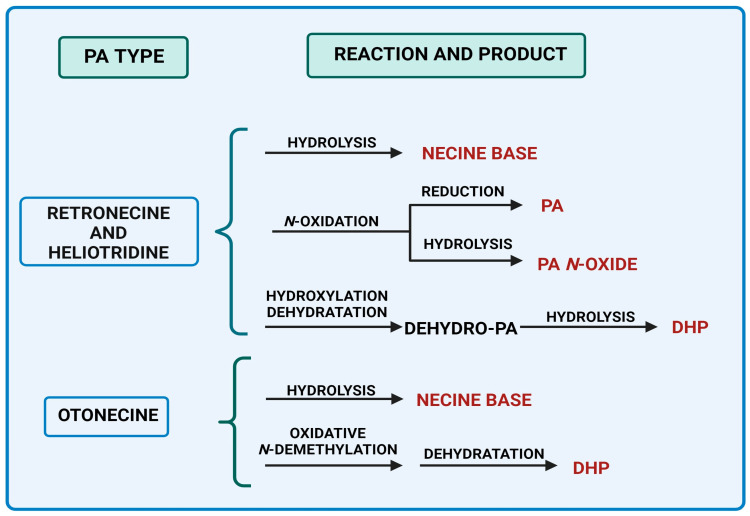
Main metabolic pathways of toxic PAs biosynthesis (BioRender, 2023).

Four principal pathways for the PAs metabolism are distinguished (Figure 3). These include hydrolysis, *N*-oxidation, and hydroxylation or *N*-demethylation [17,19,20,21].

Pyrrolizidine alkaloids of retronecine- and heliotridine-types can be synthesized as PA *N*-oxides, whereas otonecine-type PA cannot because the nitrogen is methylated in their necine base (Figure 3). PA *N*-oxides can undergo metabolic conversion into their corresponding PAs. After PAs are absorbed through the blood into tissues, some of them are cleaved by nonspecific esterases into necines and necic acids, which are non-toxic compounds [17]. Moreover, pyrrolic esters are formed through hydroxylation and dehydration (e.g., retronecine- and heliotridine-type PAs), or oxidative *N*-demethylation (e.g., otonecine-type PA) and dehydration [22]. These metabolites can bind to biomolecules like DNA and proteins, generating their adducts along with their cross-links. They are regarded as the primary metabolic triggers of the genotoxicity and carcinogenicity of PAs [17,22].

Dehydro-PA, a toxic metabolite due to its antimitotic, mutagenic, and carcinogenic properties is formed through hydroxylation and dehydration [17,22] (Figure 3). Through the hydrolysis of dehydro-PA, (±)-6,7-dihydro-7-hydroxy-1-hydroxymethyl-5Hpyrrolizine (DHP) is produced [17,22]. The pyrrole ring in DHP is involved in the rapid elimination of oxygen substituents at C7 and C9 following the production of species that react with cellular components (proteins and DNA) [23]. 

A limited number of PA derivatives originating from non-plant organisms have been isolated. For example, microorganisms like *Streptomyces* are able to synthesize pyrrolams, clazamycins, jenamidines, bohemamine, and NP25302 among others [24,25]. Pyrrolams depicted as A, B, C, and D are produced by *Streptomyces olivaceus*. The pyrrolams and clazamycins represent simpler PA structures when compared to the more typical plant-originating necine bases (e.g., retronecine) [25].

The biosynthetic origin of microbial PAs has not been extensively explored so far [2,7]. Unlike plant PAs, the biosynthesis of microbial PAs may involve non-ribosomal peptide synthetases (NRPSs). These are some of the major enzyme complexes that have been identified in numerous bacterial and fungal cells. They are responsible for the formation of the bohemamine gene cluster, facilitating the formation of the pyrrolizidine core and its methylation. They synthesize significant secondary metabolites for humans [26].

The multifunctional FAD-dependent enzyme LgnC catalyzes the biosynthesis of the new bacterial PAs, referred to as legonmycins and originating from NRPSs. It converts intermediates into pyrrolizidines using a unique mechanism within the ring structure [2,7].

Liquid chromatography (i.e., HPLC/UHPLC) is the most common technique for the analysis of PAs. For the initial stage of PAs determination, mainly C18 columns with different modifications are employed. Due to the large variety of groups within the basic structure of the molecule, diverse extraction methods and different analytical conditions are used for the determination of PAs (Table S1), depending on commercially available standards.

## 3. Plants as a Source of PAs

Within *Apocynaceae*, the genus *Parsonsia* R.Br. contains 85 distinct species of woody creepers. This genus is native to tropical and subtropical Asia and the SW Pacific. The genus *Pentalion* Voight (*Urechites* Müll. Arg.) includes only two species occurring from Florida to Central America (Caribbean). In *Parsonsia* lycopsamine, parsonsine, heterophylline, and spiranine have been identified, whereas in *Pentalion* loroquine is found [27,28].

In the *Ranunculaceae* family, the source of PAs is the genus *Caltha* L. There are rhizomatous, perennial flowering plants to which ten species have been assigned that contain senecionine. They are found in humid environments in temperate and cold regions of both the northern and southern hemispheres. Additionally, *Castilleja rhexifolia* Rydb., the only *Scrophulariaceae* member rich in PAs, contains senecionine. This species is native to W Canada, as well as NW and W central USA. This hemiparasitic perennial primarily grows in the temperate biomes [12,27].

In the *Fabaceae* family, the primary source of PAs toxicity is the genus *Crotalaria* L. with over 700 species widely distributed in tropical and subtropical regions of Africa, as well as central and E USA. The highest toxicity is displayed by *Crotalaria spectabilis* Roth, *C. retusa* L., *C. alata* Leveille, and *C. quinquefolia* L., while the lowest is found in, e.g., *Crotalaria australis* Bak. ex-Verdoorn, *C. maxillaris* Klotzsch, *C. sphaerocarpa, C. juncea* L., and *C. brevidens* Benth. [29]. Monocrotaline and spectabiline are the two types of PAs that exhibit clinical hepatotoxicity and carcinogenicity. They are found in the leguminous seeds, leaves, stems, or roots of *Crotalaria* plants. Species with monocrotaline alone are more toxic than those with spectabiline alone at the same concentrations. Monocrotaline is also the most toxic to the pulmonary vasculature. To date, there are no known species containing both spectabiline and monocrotaline and they can only have either one or the other. 

In the case of the *Asteraceae* family, PAs are dominant in two tribes with Senecioneae being the largest tribe. Almost one-third of the species in this tribe are found in the genus *Senecio* [27]. The PA-rich genera in *Asteraceae* are *Adenostyles, Brachyglotis, Cacalia, Doronicum, Emilia, Erechites, Ligularia, Petasites,* the very diverse *Senecio,* and *Tussilago*. Most of the genera and species are widespread worldwide [12].

This part of the paper focuses on reviewing species in the *Boraginaceae* family, which is unique due to the exceptional richness and diversity of its PAs [30,31,32,33]. The list of species (Table S2) is adapted from the work of El-Shazly and Wink [9], but plant nomenclature has been standardized and given according to the International Plant Names Index (IPNI) [34]. Geographical distribution and life forms are based on the Plants of the World online [27].

**Figure 4:**
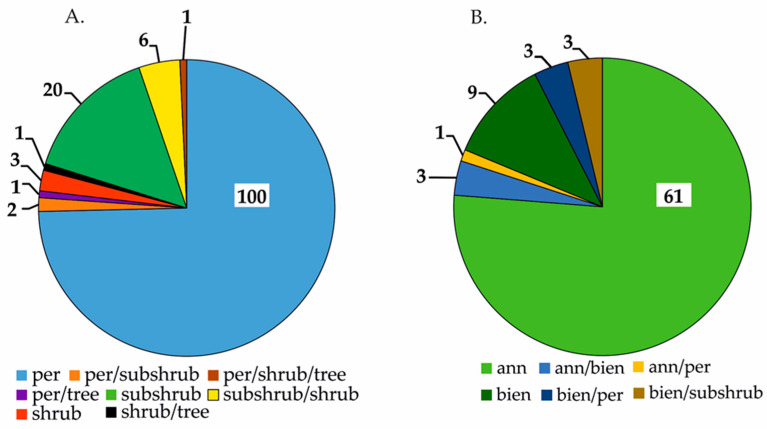
Diversity of the life forms of perennial (**A**) and annual and biennial (**B**) representatives of the PA-rich family *Boraginaceae* (expressed in number of species), where: per—perennial (herbaceous, non-woody) plant; ann—annual plant; bienn—biennial plant.

**Figure 5:**
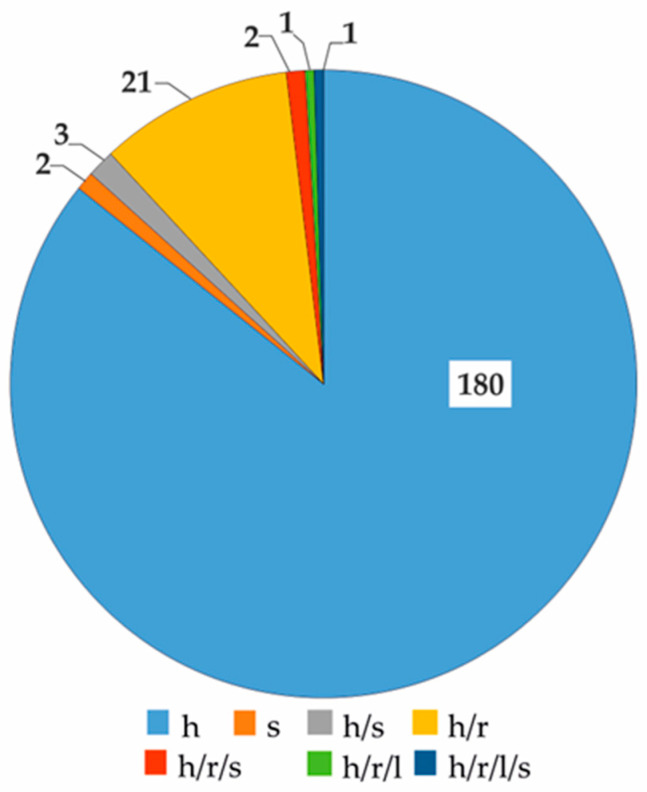
Diversity of the herbal raw materials in representatives of the PA-rich *Boraginaceae* family (expressed in number of species), where: h—herba, s—seed; r—root, l—leaf.

Within the *Boraginaceae* family, there have been 210 plants identified as containing PAs. Taxonomically, they represent 37 genera, 188 species, 8 subspecies, 12 varieties, and 2 interspecific hybrids. The genera with the highest number of known species are *Heliotropium* (45), *Echium* (17), *Amsinnckia* and *Cynoglossum* (both 16), *Symphytum* (14), and *Crypthantha* (13). The examined plant family represents three basic life forms: perennial species, including non-woody and woody representatives (Figure 4A), annual forms, and biennial forms (Figure 4B). Additionally, there is a notable presence of mixed life forms (Figure 4). In terms of the herbal raw materials offered by this plant group, the primary dominance is in herba (86%) and herba and root (10%) (Figure 5). Other raw materials have a substantially low representation (Figure 5), which can be attributed to the habitat and life forms.

**Figure 6:**
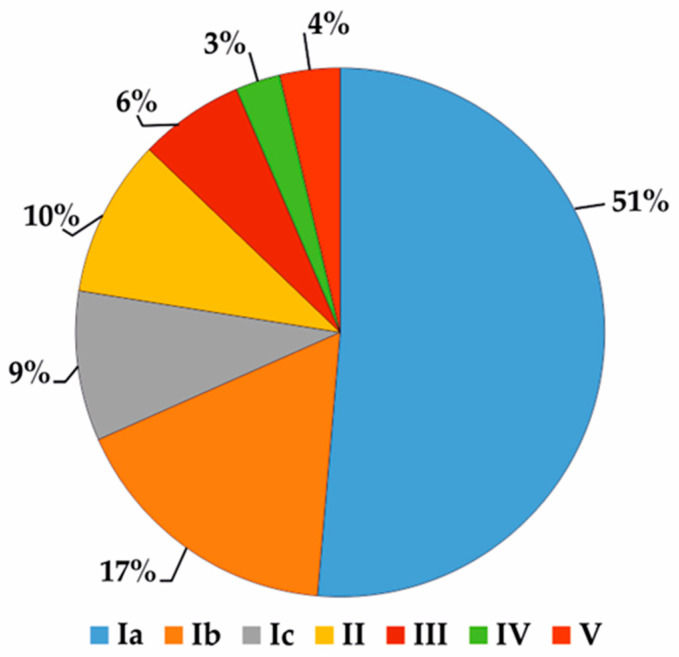
Frequency occurrence of PAs in representatives of the PA-rich *Boraginaceae* family (expressed as a percentage). Group I—rare, exclusive compounds, found in 1 (Ia), 2 (Ib), and 3 (Ic) species; Group II—moderately frequent compounds, recognized in 4 to 9 species; Group III—frequent compounds, found in 10 to 15 species; Group IV—very frequent compounds, recognized in 16 to 20 species; Group V—common compounds, found in more than 20 species. Based on the list of compounds published by El-Shazly and Wink [9].

Based on El-Shazly and Wink [9], 216 various compounds were detected in the species of the *Boraginaceae* family (Table S3). Their frequency differs significantly among species (Figure 6). Lycopsamine was detected in 60 species of the *Boraginaceae* family, e.g., genera *Amsinckia*–20, *Crypthanta*–8, and *Symphytum*–7 species. This makes it the most widespread alkaloid in the family. Intermedine, echinatine, and supinine are also commonly found, in 47 (e.g., *Amsinckia*–20, *Crypthanta*–10, *Anchusa*–2 species), 35 (e.g., *Cynoglossum*–10, *Rindera*–6, *Heliotropium*–4 and *Symphytum*–3 species), and 31 (e.g., *Amsinckia*–12, *Heliotropium*–6 species) representatives of *Boragineceae*, respectively. Rare alkaloids are often present in common species, e.g., *Echium vulgare*–6, *Cynoglossum officinale*–4, *Anchusa stigiosa*–5, *A. arvensis,* and *Borago officinalis*–2 each. To date, the only source of 7-acetyl-9-sarracinoyl retronecine is *Alkanna tinctoria*, sincamidine–*Amsinckia intermedia*, while 3’-acetylechiumine–*Amsinckia menziessi* var. *intermedia* [9].

This family comprises both commonly and easily cultivated plant species, as well as rare and endemic plants (Table S2) exhibiting a distinct combination of analyzed metabolites. Widespread species can contaminate PA crops, prompting profiling to produce reference materials that monitor their presence in bee products, herbal raw materials, food, and feed [23,35]. 

Mädge et al. (2020) [36] conducted a study in northern Germany to determine the levels of PAs and corresponding *N*-oxides in various plant species including *Echium vulgare, Symphytum* spp., *Cynoglossum officinale,* and *Heliotropium europaeum*. The results showed that the total average content of 1,2-saturated and 1,2-unsaturated PAs and *N*-oxides ranged from 357 to 32,428 mg/kg, which corresponded to 0.04% and 3.24% of the dry matter, respectively. The greatest mean amounts were discovered in *C. officinale* (32,428 mg/kg) and *H. europaeum* (15,736 mg/kg). These values exceed those found in *E. vulgare* (1330 mg/kg) and *Sympyhtum* spp. (357 mg/kg) by over 10 times. When comparing the structural diversity of PAs of four plant species, it is evident that *C. officinale* (including 57 PAs, where 35 were previously unknown) and *H. europaeum* (with 60 PAs, 29 of which were new compounds) exhibit greater diversity than *E. vulgare* inflorescences (46 PAs, with 35 alkaloids reported and 11 remaining unknown) and *Symphytum* spp. (13 PAs). Possessing a greater variety of necine bases and corresponding PAs and *N*-oxides contributes to this outcome. The alkaloids found in *C. officinale* are mostly of the heliotridine type, while other PAs comprised the 1,2-saturated necine bases trachelanthamidine and platynecine. Within *Heliotropium*, the necine base heliotridine prevails, with others assigned to trachelanthamidine and supinidine types. In the case of *E. vulgare*, the majority of PAs come from the retronecine base, except for an alkaloid that consists of a platynecine base. Heliotridine- and retronecine-type bases were observed in *Symphytum* sp. [36]. This indicates that selecting suitable profiling methods can enable the detection and identification of new alkaloids in plants that are seemingly familiar. 

In the research conducted by Stefova et al. (2022) [37], the toxicity potential of several common plant species found in Macedonia, including *E. vulgare, E. italicum* L., *S. officinale* L., *C. creticum* Mill., and *Onosma heterophylla* Griseb was analyzed. Based on the content of PAs, *O. heterophylla* and *C. creticum* were found to have the highest potential for toxicity (with levels up to 4753 mg/kg and 3507 mg/kg, respectively). This was followed by *E. vulgare* (with levels up to 1340 mg/kg), *S. officinale* (up to 479 mg/kg), and *E. italicum* (up to 16 mg/kg).

Examples of rare, endemic species in the *Boraginaceae* family are *Echium sabulicola* ssp. *decipiens* (Pomel) Klotz. and *Solenanthus lanatus* DC. E. *sabulicola* ssp. *decipiens* (syn. of *E. confusum* Coincy) growing wild in Algeria [30]. The two species studied yielded twenty-three identified PA compounds, some of which are novel phytochemicals for both the species and genus. 

The *Alkanna* genus from the *Boraginaceae* family serves as another excellent example of an endemic plant that contains high amounts of PAs [31]. It has numerous local and regional endemic species located in the southern part of the Balkan Peninsula, as well as in the Mediterranean and Irano-Turanian regions, and in subtropical areas of the world. Three species were tested (*A. primuliflora, A. graeca,* and *A. stribrnyi*) and eight PAs (7- and 9-angeloylretronecine, 7- and 9-tigloylretronecine, triangularine, triangularicine, dihydroxytriangularine, and dihydroxytriangularicine) were determined for the first time. The PAs metabolic pattern of this genus was influenced by the environmental conditions [31]. 

The representatives of the *Boraginaceae* family from the Pan-Himalaya area showed a high potential for PAs content [32,33]. Analyses of 16 species from the *Onosma* genus, 2 from *Maharanga*, 2 from *Lithospermum,* and 4 from the *Arnebia* genus were conducted. They resulted in the isolation of five PAs (supinine, europine, heliotrine, lycopsamine, and echimidine) from 24 species representing the Lithospermae tribe. The findings suggest that new PAs are present in the studied plants. 

## 4. Environmental and Food Safety

Although plants and microbes may produce secondary metabolites containing PAs, most animals lack the ability to synthesize them. Certain vertebrates, such as some amphibians, reptiles, birds, and insects, can sequester small molecule toxins from their diets [38]. The decomposed remains of these organisms along with the soil microorganisms are a potential source of PAs in the soil. Jiao et al. [39] extracted the soil samples from a tea garden using a range of solvents under ultrasonic conditions. The analysis discovered the presence of 15 PAs and their corresponding *N*-oxides in the soil.

Humans consume not only PA-containing plants (such as herbs and teas), where intake can be controlled, or plant food that may be contaminated during cultivation or harvesting. They also consume products derived from livestock or poultry fed with PA-contaminated feed (e.g., milk) and from bees that have collected contaminated pollen (e.g., honey). The taste of the two last-mentioned groups is not indicative of the presence of PAs in any way. Moreover, the growing interest among people in traditional medicine and vegetarian (or vegan) diets could lead to an increase in the number of people consuming plant material rich in PA compounds. The consumption of PAs by humans may result in both short- and long-term toxicity. 

For safety reasons, various authorities worldwide publish guidelines and recommendations concerning PAs intake based on scientific reports. In Europe, the European Medicines Agency and the European Food Safety Authority are among them. The former [40] published a statement on the contamination of herbal medicinal products with PAs. Among other matters, the issue concerning acute and chronic toxicity with dose limits was presented. The severity of contamination of co-existing plants is the most unpredictable variable affecting the amounts of PAs they contain. Also, the European Food Safety Authority [41] provided a precise statement regarding PAs content in tea, herbal infusions, honey, and food supplements. According to the European Union’s Commission Regulation, the maximum level of PA contaminants in food [42] (Table 3) refers to the sum of 21 basic PAs and the 14 additional PAs (they co-elute with some of the basic PAs) (Table 3). The lowest levels of PAs in food products were established for infants and young children (Table 4). Following rigorous good agricultural and collection practices throughout the world seems to be essential to achieve food that is free of PAs contamination and safe for consumers. 

## 5. Functions of Microbial PAs in the Environment and Their Significance for Humans

Different types of microorganisms, including endophytic and soil fungi and bacteria, produce numerous biologically active compounds from the PAs group (Table 5). However, the occurrence, configuration, and activity of microbial PAs are largely unknown. Endophytic microorganisms are often discovered for the first time in extracts from their host plants. Over time, scientists have been able to obtain these active compounds in in vitro cultures of endophytic isolates. Zhou et al. [43] isolated endophytic fungal strains from *Bruguiera gymnorrhiza*, the Chinese mangrove. During the chemical investigation of the *Penicillium* sp. GD6 culture, a novel type of PA was discovered and named penibruguieramine A. It is characterized by an unusual 1-alkenyl-2-methyl-8-hydroxymethyl pyrrolizidin-3-one skeleton. 

Another species of plant endosymbionts belongs to the genus *Epichloë* (anamorph species: *Neotyphodium*) in the *Clavicipitaceae* family [44]. A fungus named *Neotyphodium uncinatum* was isolated from *Lolium pratense* (=*Festuca pratensis*, meadow fescue) [45]. It produced lolines in the fermentation culture. Lolines are highly water-soluble alkaloids belonging to 1-aminopyrrolizidines. Their basic chemical structure comprises a saturated pyrrolizidine ring with an unusual ether bridge linking carbons 2 and 7. The fungi that produce lolines are not only endosymbionts of cool-season grasses, but they have also been identified in the tissues of *Adenocarpus* species of the *Fabaceae* family, as well as in *Argyreia mollis* of the *Convolvulaceae* family. It is noteworthy that Tofern et al. [46] did not identify loline compounds in many other species of 14 different *Convolvulaceous* genera. Lolines are known as antifeedant and repellent compounds, and are effective against many insect species at different biological stages, e.g., *Rhopalosiphum padi* and *Schizaphis graminum* (Hemiptera: Aphididae) exhibited sensitivity towards loline alkaloids [47,48,49,50]. Endophytic fungi producing PAs, such as lolines, have been found to enhance the adaptation of host plants by increasing their resistance or tolerance to predators and indirectly protecting them from environmental stressors, such as drought, heat, low light, and poor soil fertility [51]. The use of these alkaloids has also been associated with improved biomass production, tiller numbers, increased seed production, and root growth [45]. Moreover, lolines repelled insects that serve as vectors for certain viruses, which could potentially contribute to a decrease in plant infections.

These compounds also exhibit toxicity towards nematodes. The greenhouse study demonstrated that the root extracts from tall fescue grass infected with *Neotyphodium coenophialum* were nemastatic for *Pratylenchus scribneri*, a nematode that mostly infects potatoes [52]. Furthermore, the discovered loline was nematicidal at a 50 to 200 µg/mL dose. Another study showed that a lower concentration (20 μg/mL) of this PA acted as an attractant on *Pratylenchus scribneri* [53]. In a 3-week experiment [54], the seeds containing loline alkaloids were administered to mice (male and female). Animals treated with loline demonstrated no statistically significant changes in histology, hematology, blood pressure, heart rate, and motor coordination [54]. These findings may have beneficial implications in the future due to the biocontrol characteristics of fungi synthesizing PAs. 

A non-endosymbiotic fungal strain identified as *Pochonia suchlasporia* var. *suchlasporia* TAMA 87 was isolated from a soil sample collected in the vicinity of *Acer* and *Pinus* trees in Tokyo, Japan. The active fraction extracted from a solid fermentation culture of this strain contained polyhydroxylated pyrrolizidine alkaloid designated as pochonicine. This new compound is a chitinolytic enzyme system that inhibits β-nacetylglucosaminidases (GlcNAcases) of various organisms, including insects and fungi. It is expected to be suitable as an eco-friendly pesticide or fungicide (Table 5) [55]. 

Pyrrolizidine alkaloids isolated from bacterial cultures, esp. *Streptomyces*, also reveal promising properties, including antibiotic (antifungal, antibacterial), antiprotozoan, immunosuppressive, antitumoral, and antihypertensive ones. The most abundant group of bacterial PAs are bohemamines with the methyl group in the ring [26,56,57,58,59,60,61]. One of the first compounds belonging to this group of metabolites was isolated from *Actinosporangium* sp. C36,145 strain (ATCC 31127) in 1977 [56]. Several PAs from the bohemamine-type group were discovered in cultures of *Streptomyces* sp. CNQ-583 strain, an obligatory marine actinomycete bacterium (Table 5) [58].

Jiang et al. [61,62] investigated dibohemamine A and dibohemamines D–F isolated from the *Streptomyces* sp. CPCC 200497 strain. These PAs displayed potent cytotoxicity against cancer cell lines. Additionally, this microorganism synthesized quinohemanine—a quinoxalinone-bohemamine hybrid compound with anticancer potential. Further studies of the *Streptomyces* sp. CPCC 200497 strain led to the discovery of five new bohemamines J–N [63].

Another PA metabolite was discovered in the marine-derived *Streptomyces spinoverrucosus* culture. Spithioneine A and B contain a pyrrolizidine core and they were classified as bohemamine-type alkaloids with a rare ergothioneine moiety [59]. Their antibacterial (antibiotic) activities against *Pseudomonas aeruginosa* and *Bacillus subtilis* were not confirmed.

Clazamycins are compounds isolated from *Streptomyces* species (Table 5) defined as antibiotics. Clazamycin A and B, received from the *Streptomyces* MF990-BF4 culture, gave a broad range of minimum inhibitory concentration (MIC) values depending on the type of species. The MIC value was the highest for the *S. aureus* strains (even 100 µg/mL), very high for investigated *E. coli* strains (from 50 to 100 µg/mL), and low for the *P. aeruginosa* strains (MIC values were from 25 to 50 µg/mL, highlighting higher sensitivity). However, the lowest MIC values for *B. anthracis* were found to be 6.25 and 12.5 µg/mL for clazamycin A and clazamycin B, respectively [64]. In another study [65], the antibacterial effect of clazamycins on the *P. aeruginosa* strain was evaluated and the MIC values reached 20–24 µg/mL, which is comparable to the aforementioned [64]. 

**Table 5:** Studies on PAs found in microorganisms.

Type of Microorganism	Name of Species/Origin	PA	Refs.
Endophytic fungi	*Epichloe* sp. (Ascomycota: *Clavicipitaceae*), endophytes of grasses	lolines (saturated 1 -aminopyrrolizidines)	[8,44]
	*Penicillium* sp. GD6, endophyte of *Bruguiera gymnorhiza*	penibruguieramine A	[43]
Fungi	*Pochonia suchlasporia var. suchlasporia* TAMA 87 from soil	pochonicine	[55]
	*Aspergillus sclerotiicarbonarius* (IBT 28362)	sclerolizine (oxidized pyrrolizidine)	[66]
Bacteria	*Actinosporangium* sp. C36,145 (ATCC 31127)	bohemamine	[56]
	*Streptomyces* UMA-044 from sediment collected in a catfish pond, Stoneville, Mississippi, USA	bohemamine 3 NP25302	[57]
	*Streptomyces* sp. CNQ-583 from marine sediment	bohemamines 4, B, C 5-chlorobohemamine NP25302	[58]
	*Streptomyces spinoverrucosus* from marine sediment	bohemamine A, B	[59]
	*Streptomyces spinoverrucosus* SNB-032	bohemamines N; 1; 5-Cl; 5-Br dibohemamines A–C	[60]
	*Streptomyces* sp. CPCC 200497	dibohamamine A, D–F	[61]
	*Streptomyces* sp. CB02009	bohemamines 1–4	[26]
	*Streptomyces* MF990-BF4	clazamycins A, B	[64]
	*Streptomyces puniceus subsp. doliceus* NRRL 11160	clazamycin B	[67]
	*Streptomyces* sp. MA37 from Ghanaian soil	legonmycins A, B	[7]
	*Streptomyces* sp. HK10297	jenamidines A–C	[68]
	*Streptomyces spinoverrucosus*	spithioneines A, B	[59]
	*Pseudomonas fluorescens* HK 10770 from forest soil in Germany	pyreudiones A–D	[69]

Huang et al. [7] identified two new bacterial PAs, legonmycin A and B, synthesized by the *Streptomyces* sp. MA37 strain originating from the soil [2,7].

Clazamycin B, known as Antibiotic 354, and obtained from the *Streptompces puniceus* subsp. *doliceus* NRRL 11160 culture, demonstrated anticancer properties against mouse leukemia in in vitro studies. It also showed quite strong antiviral activity, reducing *Herpes simplex* types 1 and 2 even at low concentrations of 12 µg/mL (in research with primary rabbit kidney monolayers), and combated vaginal HSV-2 (in research with mice and guinea pigs) as well as HSV-1 (in research with hamsters) [67]. 

Among the secondary metabolites of the *Streptomyces* sp. HK 10297 strain, Hua et al. [68] discovered jenamidines A-C, three unknown compounds with an unusual octahydro-pyrido[1,2-a]pyrimidine skeleton. Two years later, Sinder and Dival [70] described the existence of the mixture of two diastereoisomers of these PAs through their synthesis: jenamidine A_1_/A_2._ The activity of these compounds remains poorly researched. To the best of our knowledge, there exists only one report on the antiproliferative activity of jenamidine A [68].

In studies on the defense mechanisms of microorganisms, various species of bacteria, including the *Pseudomonas fluorescens* HKI0770 strain, were isolated from the soil. This strain was resistant to predation during testing with the predatory *Dictyostelium discoideum* [11]. Dictyostelids are eucaryotic social amoebae that feed on bacteria and are commonly found in moist habitats such as the soil and leaf litter. The metabolites responsible for inhibiting *D. discoideum* were pyreudiones A–D (1–4), which are a set of pyrrolizidine diones (bicyclic PA) compounds. These studies confirmed the ability of a bacterial strain to synthesize PA compounds that are rarely found in bacteria, esp. those that do not belong to the *Streptomyces* genus. Moreover, the biosynthesis of pyreudiones 1–4 and the architecture of the NRPS gene pys (box) were analyzed [11].

## 6. Various Susceptibility to PAs

In the first half of the previous century, several studies confirmed the toxicology and carcinogenic action of PAs. Species differences exist in the impact of PAs on organisms, as observed in animal or animal cell experiments. In rats, cattle, horses, and chickens, a lack of esterase activity in their livers seems to be detrimental to high susceptibility to PAs. Herbivores, such as sheep, guinea pigs, gerbils, rabbits, hamsters, and Japanese quail, have protective mechanisms against the presence of hepatotoxic metabolites of PAs, which rely on high liver esterase activity [71,72]. Among PA-containing plants, the *Crotalaria* species are responsible for causing a significant range of tissue damage to domesticated species, like lung lesions in cattle, sheep, goats, horses, and pigs, as well as liver damage in most livestock [5].

The first serious cases of disease among humans were noticed in Asia, Africa, and South America, where medicinal herbs were most widely used [73]. Potions containing PAs used by women during pregnancy, parturition, and lactation can pose a hazard to the fetus or the suckling baby due to their passage through the placental barrier or presence in the milk.

Documented clinical studies have confirmed the toxic effects after the usage of plant products (e.g., extracts) containing PAs [4,10]. The known toxic (hepatotoxic), allergenic, mutagenic, and consequently carcinogenic effects were more commonly observed after a long-term application of such products. Herbivores have a natural aversion to plants containing PAs due to their bitter taste.

Diet significantly affects bodily condition, including the liver, the primary recipient of toxins introduced into the human body through the digestive system. Although we understand the composition of the diet, its impact cannot be estimated with certainty. The consumption of tea and herbal infusions by European populations is well known, and these are popular additives to various kinds of summer drinks like iced tea. The concentration of PAs in infusion or industrial extracts is influenced by several factors, e.g., water temperature, water-to-tea ratio, infusion time, stirring and substrate dosage form (loose leaf and tea bag), and degree of leaf fragmentation. It is worth noting that the tradition, habits, and culture of drinking such infusions provide us with continuous exposure to PAs. In addition, other components of the human diet also affect liver metabolism [14].

Dietary components appear to have a fundamental impact on the body’s ability to neutralize some toxic metabolites. Molecules of glutathione (GSH) and cysteine contain sulfur in the sulfhydryl (thiol) (-SH) group. They protect cells against free radicals and oxidative stress [74]. Metabolites of PAs may also interact with -SH groups and hence a high concentration of GSH and cysteine in the diet could potentially reduce the risk of toxic intermediate metabolites activity. The metabolic conversion of PAs produces primary DHP and secondary pyrrolic metabolites (e.g., riddelliine, monocrotaline), which leads to DNA modification and liver tumors. The hepatotoxicity of these potentially hazardous metabolites was assessed in a recent study [74] using rat primary hepatocytes with and without the addition of GSH or cysteine. It was proved that both molecules can drastically reduce the hepatotoxicity of PAs.

The gut microbiota is believed to play a crucial role in the development of various acute and chronic liver diseases, e.g., (non-)alcoholic liver disease, hepatitis virus infection, and chemical- or drug-induced liver injuries [75]. One of the potential causes of hepatotoxicity is gut dysbiosis and microbial metabolites. In an in vivo experiment, male rats were orally administered monocrotaline (90 mg/kg) once. Due to the insufficient microbiome-derived tryptophan metabolism within the gut lumen resulting in decreased activity of AhR (aryl hydrocarbon receptor)/Nrf2 (nuclear transcription factor) signaling in the liver, HSOS was induced by the applied PA. The suggested therapeutic approach for treating the PA-induced HSOS is to restore or enrich the population of specific bacteria by supplementation with specific tryptophan metabolites (AhR ligands), or to directly administer with pharmacological AhR agonists [75].

Moreover, the effective liver detoxification process depends on the overall health and condition of the liver. Considering the quality of our environment, the pace of our lives, long-distance food transport, and the high possibility of pathogen transfer, liver damage may be caused by food contaminated with mycotoxins (e.g., aflatoxins) and metals, bacterial or viral infections, long-term use of medications associated with chronic diseases, and our lifestyles in relation to alcohol consumption [14].

There are reports showing differences in the metabolism of PAs between sexes in the animal kingdom [22]. Among individuals of the same species, males exhibit greater sensitivity than females [22]. Hemangiosarcomas and hepatocellular tumors were found to affect all species/sex combinations, except for female mice as evidenced by the 2-year carcinogenicity bioassay of riddelliine in male and female rats and mice orally administered. It is concluded that aspects other than toxicokinetics are responsible for the observed species and gender specificity of the majority of toxicity effects, including the induction of liver tumors in rodents [76].

Products with PAs are distributed throughout the human gastrointestinal tract to the distant tissues and organs like the kidneys and lungs. They are mainly metabolized in the liver by various hepatic enzymes [77]. The toxicity of PAs depends on their metabolism in the liver, which is greatly influenced by the interaction of their metabolic products with various drugs taken by humans. It is challenging to anticipate the outcomes of an unknown combination of PA compositions and drug concentrations. Certain medications have been found to enhance or inhibit the activity of cytochrome P450 (CYP450). Enzymes of CYP450 are essential for the detoxification of chemicals, including alkaloids and drugs. They are mainly expressed in the liver, although they are also present in the small intestine, lungs, and kidneys, and in the placenta [78]. It is known that some CYP450 enzymes are inhibited by many common drugs, e.g., ketoconazole (Nizoral), erythromycin, trimethoprim/sulfamethoxazole (Bactrim), and ciprofloxacin (Cipro). Conversely, drugs like rifampin, carbamazepine, and phenobarbital are potent inducers [78,79]. Due to the polymorphism (genetic variability) of CYP450 enzymes, a patient’s response to a particular drug and their sensitivity to PA-containing substances may vary significantly. There is a minority of individuals known as poor metabolizers. It has been estimated that approx. 0–1% of Asians, 0–5% of Africans, and 5–14% of Caucasians lack CYP2D6 activity, which is one of the most crucial enzymes responsible for metabolizing almost 80% of drugs used [80].

The toxicity of PAs can be influenced by age. Fetuses, neonates, and children are considerably more sensitive to this group of compounds than adults. In the past, Rasenack et al. [81] presented one of the best-documented cases of a newborn suffering from hepatic veno-occlusive disease, known as hepatic sinusoidal obstruction syndrome (HSOS), typical of PAs poisoning. Cases of severe liver injury have been observed in patients who consumed small amounts of PAs over a prolonged period of time, e.g., daily consumption of herbal tea of unknown origin. Pregnant women are esp. vulnerable to this effect. Caution must be taken because the concentration of PAs may significantly exceed the expected norm for food contamination.

## 7. Hepatotoxicity

Among the key and extensively distributed hepatotoxins in plant species are PAs. These substances have the potential to disrupt the normal redox state in the microenvironment of the liver. Exposure to PAs resulted in an increased generation of free radicals, while GSH production was depleted. Glutathione plays a vital role in countering oxidative stress by facilitating detoxification [82]. Reactive PA metabolites are responsible for the induction of hepatotoxicity and PA-induced liver damage has been reported worldwide [83].

The consumption of products containing PAs can lead to a severe health condition named HSOS. As a result of toxic damage to the hepatic sinusoidal endothelial cells (HSECs), liver enlargement, ascites, and hyperbilirubinaemia occur [84,85]. In the absence of medical treatment, severe consequences of HSOS appear, including fibrosis, cirrhosis, necrosis, and eventually death [86,87,88]. The ingestion of the *Gynura japonica* (Thunb.) Juel (*G. japonica*) is the main cause of HSOS in China. Unintentional consumption of this herb accounts for 50–89% of all reported HSOS cases. A high intake of PAs from naturally sourced has been linked to over 8000 cases of chronic liver injury [84].

The metabolically competent HepG2, a human hepatoma cell line, was used to bioactivate PA. HepG2 cells are capable of the excretion of toxic and mutagenic pyrrole metabolites. The PA-induced disturbances in mitosis may contribute to the formation of micronuclei. Europine, riddelliine, and lasiocarpine induced micronuclei in HepG2 cells [89].

Hepatic sinusoidal endothelial cells were stimulated by the senecionine. Dynamic changes were observed in the damaged proteome, and PA induction resulted in TSP1 (thrombospondin 1) overexpression. TSP1 overexpression was further confirmed in liver samples and human HSECs from patients with PA-induced HSOS. Furthermore, the study revealed that dehydropyrrolizidine alkaloids covalently modify TSP1 in HSECs and mouse livers after treatment with senecionine, leading to the formation of a protein pyrrolo-adduct. These results provide insight into the initial alterations that occur in HSECs after exposure to PA and highlight the link between TSP1 overexpression and the development of PA-induced HSOS [85].

The conducted studies have confirmed the significant hepatotoxicity induced by PAs, and therefore new strategies are needed to alleviate the liver dysfunction. Hyperoside, a natural flavonoid, has exhibited a protective effect on the liver damaged by PAs. Its beneficial properties mainly include the alleviation of hepatotoxicity and regulation of mitochondrial homeostasis [88]. Furthermore, the promotion of TFEB (transcription factor EB (TFEB)-peroxisome proliferator-activated receptor-γ coactivator-1-α (PGC1α) pathway) nuclear translocation was caused by the inhibition of mTORC1 (mechanistic target of rapamycin complex 1) activity after hyperoside application. This process is linked to both the autophagy-lysosomal pathway and mitochondrial biogenesis. In addition, studies have demonstrated the hepatoprotective properties of hyperoside in a number of medical conditions including paracetamol-induced liver damage, carbon tetrachloride hepatotoxicity, and cisplatin-induced liver damage [88].

Liver damage may occur with the use of drugs, dietary supplements, herbal products, or xenobiotics. Recent studies have demonstrated that PAs consumption can trigger drug-induced liver injury (DILI), which can be associated with damage to the mitochondria in the liver cells [90,91]. Tests conducted on HepaRG cells indicated negative effects of some PAs, including retrorsine, monocrotaline, retronecine-retronecine-type, and plathphylline-platynecine-type on the liver cells. They confirmed an increase in oxidative stress level, calcium concentration, and endoplasmic reticulum dysfunction, as well as a decrease in mitochondrial membrane potential and neutral lipid metabolism [91]. Additionally, exposure of hepatic RNA to retrorsine in mouse liver microsomes resulted in adenosine and guanosine adduction. Significant alterations in the properties and metabolism of the adducted RNA were discovered, including RNAse susceptibility [83].

Hepatocellular carcinoma (HCC) occurs in the context of a fibrotic or cirrhotic liver after hepatitis C or B viral infection. The progress in using genomic and proteomic data to comprehend the molecular development of HCC resulted in the possibility of providing new clinical benefits to patients. Moreover, the genetic aberrations of HCC show considerable heterogeneity due to differences in ethnicity and environmental susceptibilities [92]. Some proteins can bind with pyrrole metabolites, forming pyrrole-protein adducts [93]. Mutations of six human pyrrole-protein adducts in HCC genomes showed significant cytosine-to-adenine transversion with a mutation rate of 32%. It was found that retrorsine-induced DNA addition was the trigger for the activation of cancerous liver progenitor cells, which initiate hepatocarcinogenesis [77].

Genetically modified cells expressing the cytochrome P450 enzymes were employed to examine the effect of PAs. To investigate cytotoxicity, modified HepG2 cells with overexpressed cytochrome P450 enzymes (mostly CYP3A, but also CYP2B subfamilies) were used [94]. In HepG2-CYP3A4 cells, a reduction or inhibition of cell viability was observed in monoesters of indicine (92%), lycopsamine (85%), and europine (77%), and a significant reduction in heliotrine (44%) monoester was seen. Prolonged exposure to PAs could potentially impact the cytotoxicity of HepG2-CYP3A4 cells. A correlation between increased cytotoxicity and higher concentrations of PAs was proved.

Indicine and lycopsamine displayed the lowest cytotoxicity, and EC_50_ values remained unmeasurable even after 72 h of exposure. In contrast, europine and monocrotaline had EC_50_ values ranging from 200 to 500 µM after 72 h. Lycopsamine and indicine caused a slight decrease in cell viability in a concentration-dependent manner. In contrast, europine and heliotrine exhibited a more pronounced reduction in viability after 72 h of exposure. Lasiocarpine and seneciphylline showed the most significant cytotoxicity with a reduction in cell viability to 21% at 40 µM and 24% at 100 µM, respectively. It is noteworthy that monocrotaline, as a cyclic atypical diester, caused only mild cytotoxicity (77%) at 500 µM [94].

Rat hepatocytes provide an ideal model for studying PA-induced cytotoxicity. All diesters and molecules with open rings have very high cytotoxicity, excluding monocrotaline. Cytotoxicity of PAs is time- and concentration-dependent. For example, in the case of retrorsine and seneciphylline, a short preincubation period makes the subsequent 24 h incubation less toxic [95].

Various toxicokinetic parameters of PAs were elucidated. The transport rates of differently structured PAs (monoesters, open-chained diesters, and cyclic diesters) were studied in in vitro models [96]. It was shown that PAs are metabolized by liver microsomes in a structure-dependent manner and, to a lesser extent, by lung microsomes. After applying lasiocarpine and monocrotaline, apoptosis in the alveolar basal epithelial adenocarcinoma A549 cell line was detected. Such findings may contribute to a better understanding of molecular processes leading to the effects of PAs observed in in vivo studies [96].

**Figure 7:**
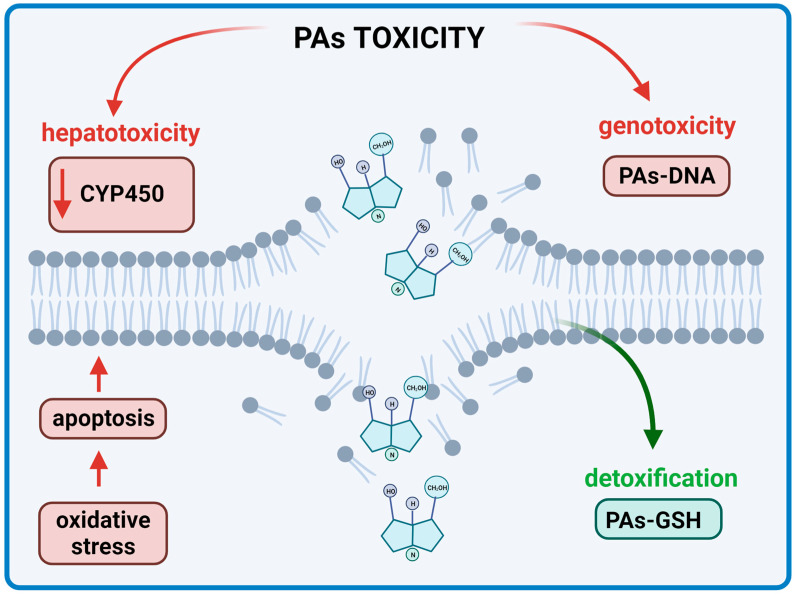
Toxicity effects of Pas on the human body (BioRender, 2023).

The toxicity induced by PAs is realized by different pathways and sequences of reactions [23] (Figure 7). The potency of toxicity depends on several factors. Firstly, the bioavailability of the alkaloids after oral administration is important. Secondly, how the transport of the substance through the intestine takes place in addition to absorption is crucial. The absorption pathway in the liver and the activation of the complicated metabolic pathways should also be emphasized [96].

## 8. Pyrrolizidine Alkaloids and Their Positive Activities

Among numerous interesting activities of PAs, their anticancer and antimicrobial properties seem to be the most promising for further applications [4,10].

### 8.1. Anticancer Properties

The severity of cancer was estimated by statistics which show that in 2020 approx. 19.3 million people were affected, and half of these cases resulted in fatalities [97]. Moreover, this figure is predicted to increase by 47% by 2040, leading to a global health challenge.

Existing therapeutic options such as chemotherapy have had only limited success due to unwanted side effects on the patients combined with the extremely high costs. Therefore, scientists have been in search of new therapies [98]. An important target in cancer therapy is the regulation of programmed cell death (PCD), which can be divided into two types, I—called apoptosis, and II—named autophagy. Apoptosis is known to be driven by two major pathways: the receptor pathway (extrinsic) and the mitochondrial (intrinsic) pathway. Despite the pathway, they are dependent on the activation of caspases [99]. The extrinsic apoptosis pathway is associated with the interaction of appropriate ligands with receptors located on the surface of cell membranes. The membrane receptors involved in this pathway are tumor necrosis factor (TNF) receptors 1 and 2, TRAIL DR4 receptors, and Fas receptors, which recognize their specific death ligands such as TNF ligand, Fas ligand (FASL) or TNF-related apoptosis-inducing ligand (TRAIL). Upon the binding of the ligand to the appropriate membrane receptor, the intracellular region of the death receptor recruits an adapter molecule through the TNF receptor-associated death domain (TRADD) or FAS-associated death domain (FADD). In the next step, inactive procaspase-8 is attached to the death-inducing signaling complex (DISC) and becomes activated. Then, caspase-8 initiates the executive phase of the apoptosis process by activating caspase-3 and -7 [100,101,102,103].

The intrinsic apoptosis pathway can be activated by many factors, including cytokines, hormones, hypoxia, increased levels of free radicals, lack of factors determining cell growth, DNA damage, increased Ca^2+^ concentration in the cell cytoplasm, and ionizing or ultraviolet (UV) radiation. As a result of the stressor, the permeability of mitochondrial membranes increases and pro-apoptotic proteins like NOXA, BAX, and PUMA are activated, which initiates the apoptosis process. The change in mitochondrial membrane permeability is associated with the opening of the mitochondrial permeability transition (MPT) pore. The effect of pore opening is a decrease in membrane potential. This phenomenon causes the release of pro-apoptotic proteins (e.g., cytochrome c, HtrA2/Omi, Smac/Diablo) from the mitochondria into the cytoplasm. Cytochrome c combines in the cytoplasm with the APAF1 factor, which then attaches to the caspase recruitment domain (CARD) of procaspase-9. A complex called apoptosome is formed, which activates caspase-9. Caspase-9 activates effector caspases-3 and -7 and the apoptosis process is induced [100,101,102,103].

Autophagy, type II PCD, is involved in the formation of autophagosomes, which further fuse with lysosomes to form acidic vesicular organelles, including autolysosomes [99]. Autophagic activation can play a protective role in the early stages of cancer progression by activating pro-autophagic genes and blocking anti-autophagic genes in oncogenesis. The mechanism of autophagy is quite complex. In brief, it is initiated by either the inhibition of the mechanistic target of rapamycin (mTOR) or activation of 5′ AMP-activated protein kinase (AMPK) [99,104].

For medical applications, an ideal PA would have a high tumor inhibitory effect coupled with low hepatotoxicity. *Liparis nervosa* belongs to the *Orchidaceae* family and is found in many parts of China. It is used in traditional herbal medicine for skin inflammation, swelling, and the detoxification of snake venom [105]. Chen et al. [17] conducted a study on the antitumor effect of PAs isolated from *L. nervosa* on HCT116 human colorectal cancer cells. It was concluded that among the eight tested nervosine derivatives, chloride-(*N*-chloromethyl nervosine VII) showed the most significant antitumor activity at IC_50_ = 32.88 ± 1.6 μM. The potential mechanism for the anticancer effect of chloride-(*N*-chloromethyl nervosine VII) may be the activation of the internal apoptosis pathway. The results demonstrated a dose-dependent increase in cytochrome c expression, leading to increased cleavage of procaspase-3 and -9. Activated caspase-3 cleaves the poly(ADP-ribose) polymerase (PARP) protein, which is involved in DNA repair processes and the regulation of transcription and intracellular signaling pathways, as well as processes of metabolism and cell death. Nervosine VII has been shown to participate in the activation of the autophagy process. This molecule was found to increase the expression level of LC3-II and Beclin 1 while decreasing the expression of p62 in HCT116 cells. The effect of nervosine VII on the process of autophagy and apoptosis is related to the activation of the signaling pathway of the mitogen-activated protein kinase (MAPK) cascade, involved in the regulation of many cellular processes. According to the experiments performed, nervosine VII caused the concentration-dependent phosphorylation of extracellular signal-regulated kinases 1 and 2 (ERK1/2), p-p38, and p-c-Jun N-terminal kinase (JNK) in HCT116 cells [106].

In another study, the saturated PA nervosine VII was investigated for its effect on PCD I and II in HCT116 human colorectal cancer cells [107]. The HCT116 cells were treated with nervosine VII at 6.25, 12.5, 25, 50, and 100 μmol·L^−1^ for 24, 48, and 72 h. The results showed that this PA inhibited the growth of HCT116 cells in a time- and concentration-dependent manner with an IC_50_ value of 11.72 μmol·L^−1^ at 24 h.

**Figure 8:**
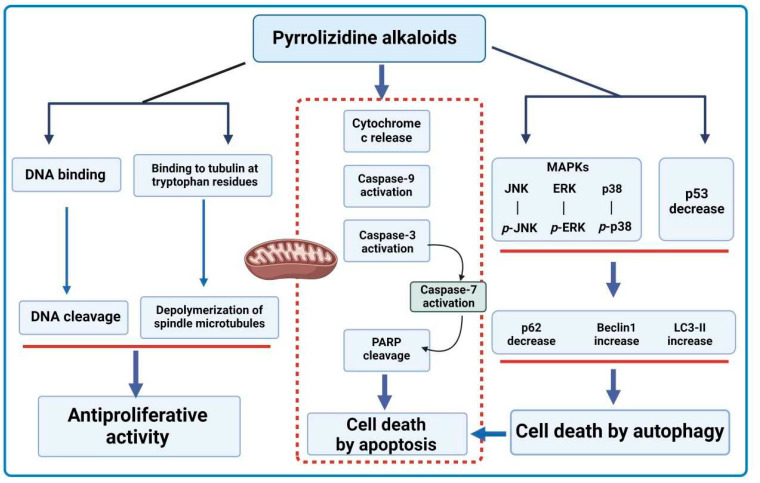
Simplified scheme of the mechanism for anticancer activity of PAs (BioRender, 2023).

The mechanism of action showed that type I PCD intrinsic pathway involved caspase 3, 7, and 9 [4]. Autophagy induction by nervosine VII was proved by the detection of autophagic biomarkers such as microtubule-associated protein light chain 3 (LC3)-II. LC3-II level was shown to have increased after a cotreatment with nervosine VII. The data suggest that this PA increased autophagosome accumulation. The effect of this PA on the crosstalk between apoptosis and autophagy was studied by inhibiting autophagy. The inhibitory effect led to the activation of nervosine VII-mediated apoptosis. It was concluded that the induction of autophagy and apoptosis by nervosine VII is a simultaneous rather than a successive process [107]. Other mechanisms that induce apoptosis and autophagy, such as the MAPK signaling pathway, have also been activated by nervosine VII through the phosphorylation of ERK1/2, p38, and JNK in a dose-dependent manner [107] (Figure 8).

*Heliotropium indicum* is one of many plants of the *Heliotropium* genera that have been used as a source of herbal medicine in some regions of Asia [108,109]. The extracts of this plant contain many PAs important from a therapeutic point of view. One such PA is indicine-*N*-oxide, especially because this molecule lacks the significant hepatotoxicity other pyrrolizidine-free bases possess. Instead, it shows more of an anticancer property, with the balance between its hepatotoxicity and anticancer property dependent on the reactive pyrrole metabolites derived from the alkaloids [110]. Appadurai and Rathinasamy [111] studied the effect of indicine-*N*-oxide isolated from *H. indicum* on cancer cell lines such as B16 (mouse melanoma), walker 256 (carcinosarcoma in rats), P388, and L1210 (murine leukemia). Indicine-*N*-oxide was able to inhibit cell proliferation in a concentration-dependent manner (Table 6). Studies have shown that the inhibitory effect of indicine-N-oxide on cell proliferation may be due to blocking the cell cycle at the stage of mitosis. Higher concentrations of this compound (300 µM) reduced the length of spindle microtubules in HeLa cells, and in some cells induced their complete depolarization. Indicine-*N*-oxide has been shown to bind to tubulin protein near tryptophan residues and inhibit tubulin polymerization in vitro [4,111] (Figure 8).

Culvenor [112] examined the tumor inhibitory effects of 18 PAs (3 *N*-oxides) and several derivatives in in vivo models of different cancers: adenocarcinoma 755, lymphoid leukemia L1210, sarcoma 180, walker 256, and plasma cytoma 1. The most inhibited tumor was walker 256 (intramuscular) exhibiting complete destruction with heliotrine at a dosage of 125 mg/kg. Adenocarcinoma 755 was strongly inhibited by monocrataline-type bases. Sarcoma 180 was inhibited by heliotrine, lasiocarpine, monocrotaline, and the chloro-derivative. Lasiocarpine showed significant activity against walker 256 (subcutaneous). None of the PAs showed significant activity against leukemia L1210 and plasma cytoma 1.

Apart from the antitumor effects, hepatotoxicity in the animal models was also observed. The antitumor activity was associated with the alkylating ability of the allylic ester system (the same functional region in the molecule associated with hepatotoxicity). It was concluded that the toxicity of the *N*-oxides overshadowed the antitumor activity. This study showed that more work needs to be put into finding the balance between the antitumor effects and hepatotoxicity of PAs if we want to use them in clinical trials [98].

As indicine-*N*-oxide showed promising results as an anticancer agent in in vitro and in vivo assays, clinical trials were conducted for acute leukemia; however, due to severe hepatotoxicity, the compound was not further tested [113,114]. One clinical trial investigated the effect of indicine-*N*-oxide on patients with progressive acute leukemia refractory to standard chemotherapeutics. Ten patients took part in the study, six of whom had acute lymphocytic leukemia (ALL), three had acute myelocytic leukemia (AML), and one had acute myelomonocytic leukemia (AMML). The ages of the patients ranged from 4 to 67 years old. A lyophilized form of indicine-*N*-oxide, at a dose of 3.0 g/m^2^, was administrated intravenously for five consecutive days. Based on the outcome, two patients achieved complete remission, while one patient experienced partial remission. As evidenced by these findings, indicine-*N*-oxide shows potential in treating ALL and AML. Most patients suffered from a range of side effects, such as mild nausea, mild diarrhea, maculopapular rash, and jaundice. It is clear that indicine-*N*-oxide has anticancer activity; however, more intensive investigations need to be carried out to find the full potential of this PA or how it can be modified to remove the unwanted side effects [10,114]. Studies on other anticancer activities of PAs from plant sources are presented in Table 6. The prevailing microbial source of PAs is the genus *Streptomyces*. Subsequently, Table 7 shows the in vitro antimicrobial activities of this strain. However, the in vivo studies are extremely limited. A single report employs clazamycin A sourced from *Streptomyces* no. MF990-BF4 (closely related to *S. violaceorectus* and *S. cinereoruber*) as an example of PAs. The experiment was performed in mice inoculated with leukemia L-1210 cells at daily intravenous doses of 12.5–100 µg for 10 days. The intravenous acute LD_50_ of clazamycin A was in the range of 50–100 mg/kg. As a result, a 130% increase in the survival time of mice was demonstrated [64].

**Table 6:** Anticancer activity of PAs isolated from plant source.

Compound	Plant Source	Cell Line	Dose	Effect	Ref.
In Vitro					
Indicine-*N*-oxide 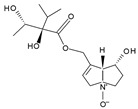	*Heliotropium* *indicum*	B16 (mouse melanoma), walker 256 (carcinosarcoma in rats), P388, and L1210 (murine leukemia)	46–100 μM	Inhibition of cell proliferation	[111]
7-angeloyl heliotrine 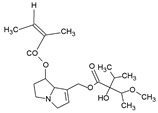	*Heliotropium* *subulatum*	Chinese hamster V79 cells	100, 50, 30, 20, 10, 5, 3 and 1 µg/mL for 5 days	Cytotoxic effect at IC_50_ concentrations of 10 and 5 mg/mL	[115]
Ligulachyroine A 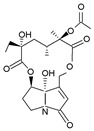	*Ligularia* *achyrotricha*	human leukemia cell (HL-60) and human hepatoma cell (SMMC-7721)	The cells were treated with various concentrations for 48 h	A moderate cytotoxic effect at IC_50_ values of 12.17 µg/mL and 11.88 µg/mL, respectively	[116]
In Vivo					
Pyrroline bis(carbamate) 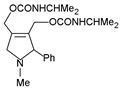	synthetic analogue of indicine-*N*-oxide	P388 lymphocytic leukemia	100, 50, 25, and 12.5 mg/kg by injection of a solution in distilled water. 5× daily doses were administered beginning 24 h after tumor inoculation	Antileukemic effect of 225% T/C at 100 mg/kg (the most active among 5 other analogues of indicine-*N*-oxide)	[110]
7-angeloyl heliotrine 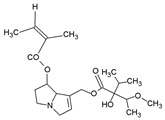	*Heliotropium* *subulatum*	ICR albino mice implanted with Sarcoma 180 (1 × 10^−6^ cells/0.1 mL ascitic fluid)	Test group: 50–100 mg/kg/day of test crude extracts + 5–10 mg/kg/day of test compounds; control group: saline only	Maximum inhibition of 41.7% at 5 mg/kg/day (the highest inhibition among five other PAs)	[115]

The cytotoxic nature of PAs renders them potential anticancer agents. However, two issues seem to be troublesome. Firstly, they display a deficiency in specificity to cancer cells. Secondly, they can convert to their toxic intermediates. Kurimoto et al. [117] have proposed a new strategy to overcome these limitations. Biocompatible artificial metalloenzymes containing ruthenium and gold assisted in the conversion of PAs into their toxic intermediates, DHPs, through an organometallic reaction, resulting in bond formation. The synthetic precursor was successfully converted to the backbone of DHPs. It was shown that the synthesized DHP 12 from precursor 11 had the same reactivity towards proteins as natural DHPs. Moreover, the toxicity of DHP 12 was confirmed by the significant decrease in EC_50_ towards various cancer cell lines (HeLa, PC3, A549, and SW620) when compared to its precursor [117].

### 8.2. Antimicrobial Properties

The coevolution of antimicrobial agents and microorganisms, described as the ‘red queen effect’, has resulted in microbial resistance to classical antibiotics, and the rapid progression of this phenomenon requires immediate solutions for the treatment of infectious diseases. Therefore, the potential of phytochemicals such as PAs has been studied thoroughly throughout the years [118]. Studies on the antimicrobial activity of PAs [4,10] are presented in Table 8.

The *Heliotropium* genera are known to be a source of a plethora of PAs with medicinal properties. Europine along with three other PAs were isolated from *Heliotropium ellipticum.* Europine showed the highest antimicrobial activity among all isolated PAs, inhibiting *Escherichia coli* and *Enterobacter cloacae* (zone of inhibition: approx. 10–12 mm). It also exhibited antifungal activity against *Aspergillus flavus*, *Drechslera tetramera,* and *Fusarium moniliforme* (zone of inhibition: approx. 7–11 mm) [4,120].

**Figure 9:**
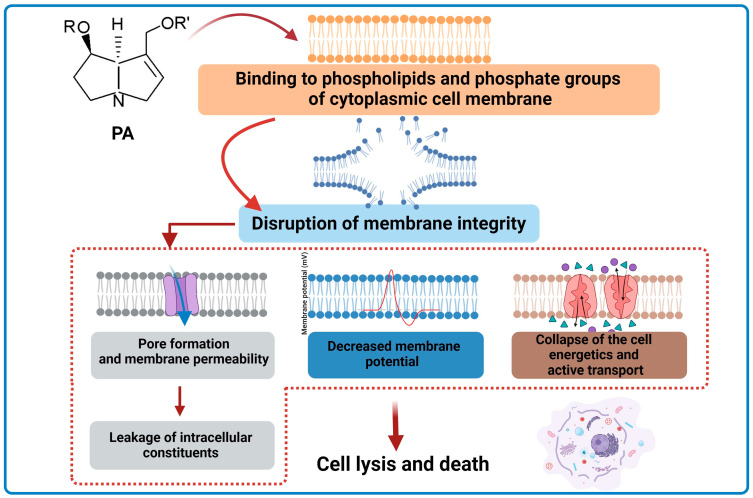
Simplified scheme of the mechanism for antibacterial activity of PAs (BioRender, 2023).

In a study by Li et al. [119], the antimicrobial activity of a novel synthesized PA-1 was investigated. The antimicrobial effect was tested against six bacterial and two fungal species. The results of the investigation showed that PA-1 is effective against *E. coli*, completely killing the bacteria within 8 h. The research showed that PA-1 has a stronger antimicrobial effect against Gram-positive bacteria compared to Gram-negative bacteria and fungi. The observed phenomenon may result from the mechanism of action of PA-1. It has been shown that PA-1 reduces the membrane potential and changes membrane permeability in *E. coli* and *S. aureus* by damaging cytoplasmic membranes, which consequently leads to the leakage of intracellular components. The influence of lecithin and phosphate on the antibacterial activity of PA-1 was also examined. Different results were obtained depending on the bacterial species. Lecithin and phosphate groups were targeted by the antibacterial activity of PA-1 only in *S. aureus*. In summary, PA-1 exhibits antibacterial activity in Gram-positive bacteria by acting on phospholipids and phosphate groups of cell membranes and then damaging cell membranes [119] (Figure 9).

The effects of PAs from *Senecio jacobaea* on the growth of nine plant-associated fungal strains (five strains of *Fusarium oxysporum*, two of *Fusarium sambucinum,* and two of *Trichoderma* sp.) were analyzed [123]. A mixture of PAs containing senecionine, seneciphylline, jacobine, and jaconine (at 12, 22, 24, and 24%, respectively) was highly effective against all fungi strains at concentrations between 0.33 mM and 3.33 mM for each individual PA. The most inhibited fungus belonged to the genus *Trichoderma* [4,10,123].

Research was conducted on the effect of usaramine on biofilm inhibition in *Pseudomonas aeruginosa* and *Staphylococcus epidermidis* [122]. It was found that this PA at a dose of 1 mg/mL inhibited the biofilm formation by *S. epidermidis* by approx. 50% without killing the bacteria. The tested PA had no effect on the biofilm formation by *P. aeruginosa*. The mechanism of this action has not been investigated [122].

The antimicrobial activity of PAs is not just limited to bacteria and fungi. Monocrataline extracted from *Crotalaria retusa* seeds when applied at a dose of 1 mg/mL showed significant inhibition of 74% of the parasitic protist, *Trichomonas vaginalis*, responsible for trichomoniasis, the most common non-viral sexually transmitted infections. Although retronecine did not show any activity against *T. vaginalis*, its semi-synthetic derivative azido-retronecine showed a 10% higher inhibition capacity than monocrataline at a dose of 1 mg/mL [10,122].

### 8.3. Potential Applications of PAs and Future Perspectives

Based on the literature, PAs possess many potential applications. Some of them are presented in Table 9.

Both plants and microorganisms appear to provide an endless source of natural substances. In the past, bioactive compounds were mainly identified from plant extracts, esp. from those plants known for their medicinal properties over centuries. Pyrrolizidine alkaloids, which are compounds with important biological activities, have been associated with the plant kingdom for many years. The pyrrolams, clazamycins, and penibruguieramine A are rare examples of PAs isolated from microorganisms. In contrast to the numerous plant-derived PAs, these recently discovered compounds are distinctive, have exceptional molecular structures and synthesis mechanisms, and consistently present alternative options in the scientific field [25,43,59].

Studying the biological activity of compounds with uncommon or unique structures leads to the identification of novel cellular mechanisms, including those affecting pathogenic microorganisms and cancer cells. Research on microbial PAs suggests previously unknown mechanisms of action in the natural environment. Huang et al. [7] studied legonmycins biosynthesis involving NRPSs. Further research indicated that a gene cluster, *pxaAB* and its homologues, have been found in over 90 bacterial strains that possess the capability of synthesizing bacterial pyrrolizidine derivatives [24]. In the future, creating the optimal conditions to induce the synthesis of novel PAs may lead to the discovery of substances displaying uncommon biological potential.

## 9. Conclusions

Pyrrolizidine alkaloids are a group of secondary metabolites synthesized by plants and microorganisms. Although PAs pose potential health risks to humans and some animals by causing toxicity, esp. hepatotoxicity, they also demonstrate beneficial pharmacological properties, e.g., antibacterial, antifungal, antivirus, anticancer, and anti-inflammatory ones.

The application of plants containing PAs in traditional medicines and in the food industry determines the necessity to further develop the specific knowledge concerning the pharmacology and toxicology of PAs and to formulate national and international standards for their quantity in herbs and food products or supplements. The main difficulty in the standardization of PA applications involves a lack of large-scale clinical evidence allowing the evaluation of a toxic dosage threshold for a whole range of various PAs. It is expected that the determination of the precise safety limits for PAs in humans will expand their potential in the food and medical sectors.

The employment of analytical methods will support research first, on the isolation and bioactivity of PAs; second, on reducing the toxicity of PAs in everyday use; and finally, on the modification of structures to achieve higher selectivity to cope with selected health problems.

In the presented work, we have tried to demonstrate that PAs, despite their toxicity, have beneficial biological properties, and the prospects for using them in medicine and the pharmaceutical industry are promising. Further detailed research on the mechanisms of action of selected PAs may contribute to the development of the new therapeutic strategies for oncological patients. Particular attention should be paid to the potential application of PAs in the treatment of cancer, including colorectal cancer. The development of mechanisms to direct the hepatotoxic effect of PA against cancer cells would also enable the potential treatment of liver tumors. These alkaloids may also be widely used in the microbiological industry as compounds that inhibit the biofilm of specific bacterial strains or as antifungal compounds. Their antiviral activity (including HIV) makes PAs an interesting subject of synthetic research.

## Figures and Tables

**Table 1:** Characteristics of types of necine bases.

Characteristics	Retronecine	Heliotridine	Otonecine	Platynecine
Ring	bicyclic	bicyclic	monocyclic	bicyclic
Unsaturated 1,2-double bond	+	+	+	−
Toxicity	+	+	+	−

**Table 2:** Types of PAs. Names of exemplary PA structures are underlined.

Combination of Necine Base and Necic Acid Forming Type of PA	Examples of PA	Examples of PA Structures
senecionine	senecionine, platyphylline, rosmarinine, nemorensine	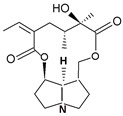
triangularine	triangularine, sarracine, macrophylline	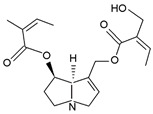
lycopsamine	lycopsamine, uplandicine	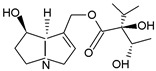
monocrotaline	monocrotaline, aucherine	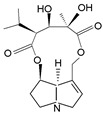
phalaenopsine and ipanguline	nervosine VII, nervosine I, ipanguline B	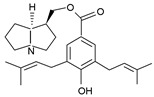
triangularine and lycopsamine	echimidine, heliosupine, scorpioidine	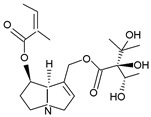
simple	acetyllaburnine, 7-acetylretronecine	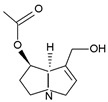
unusual	madurensine, labunamine, tussilagine	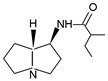

**Table 3:** List of basic and additional PAs according to Commission Regulation (2023/915).

Basic PAs	Additional PAs
intermedine/lycopsamine	indicine
senecionine/senecivernine	echinatine
seneciphylline	rinderine
retrorsine	integerrimine
echimidine	heliosupine
lasiocarpine	spartioidine
europine	usaramine
heliotrine	with their *N*-oxide forms
with their *N*-oxide forms	
senkirkine	

**Table 4:** Maximal levels of pyrrolizidine alkaloids in food according to Commission Regulation (2023/915).

Products	Max Level of PA [µg/kg]	Exceptions	Max Level of PA [µg/kg]
Tea, flavored tea, and herbal infusions (liquid product) for infants and young children	1	−	−
Tea and flavored tea (dried product)	150	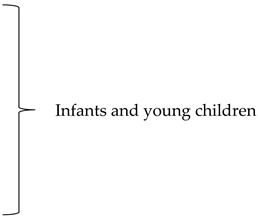	75
Herbal infusions (dried product) and ingredients used for herbal infusions (dried products)	200		
but of rooibos, anise, lemon balm, chamomile, thyme, peppermint, lemon verbena and mixtures exclusively composed of them	400		
Cumin	400	−	−
Food supplements containing botanical preparation including extracts	400	Pollen-based food supplements Pollen and pollen products	500
Dried herbs	400	Borage, lovage, marjoram, and oregano (dried product) and mixtures exclusively composed of them	1000

**Table 7:** In vitro studies on anticancer activity of PAs from *Streptomyces* strains.

PA	Microorganism Source	Cell Line	Assay Type	Effect	Ref.
derivatives of bohemamines: a quinoxalinone-bohemamine hybrid compound quinohemanine (1), 1-methyl-2(H)-quinoxalin-2-one (2)	*Streptomyces* sp. CPCC 200497, soil isolate from China	liver cancer cell line HepG2	as in sulforhodamine B (SRB) method	moderate cytotoxicity against HepG2 with IC_50_ 65.9 and 52.5 μM for (1) and (2), respectively)	[62]
dibohemamine F	*Streptomyces* sp. CPCC 200497	cancer cell lines of lungs A549 and liver HepG2	as in SRB assay	cytotoxicity against cancer cell lines A549 and HepG2 with IC_50_ of 1.1 and 0.3 μM, respectively	[61]
dibohemamines D and E	*Streptomyces* sp. CPCC 200497	cancer cell lines A549 and HepG2 of lungs and liver, respectively	as in SRB assay	moderate cytotoxicity against both cancer cells; for dibohemamines D and E IC_50_ from 7.3 for HepG2 to 39.2 for A549, respectively	[61]
dibohemamines B and C	*Streptomyces* *spinoverrucosus* marine-derived	non-small cell lung cancer cell line A549	not mentioned	dibohemamines B and C extremely potent against A549 with IC_50_ values of 0.140 and 0.145 µm, respectively	[60]
dibohemamine C	*Streptomyces* *spinoverrucosus* marine-derived	non-small cell lung cancer cell line HCC1171	not mentioned	IC_50_ value of 1.2 µm	[60]

**Table 8:** Studies on antimicrobial activity of PAs. PA dose is the dose applied in disc diffusion test. “-” not applicable.

Microorganism	PA	PA Dose	Observations	Antimicrobial Activity	Ref.
Bacteria					
*Bacillus anthracis*	heliotric acid	2 mg/mL	Zone of inhibition 14.61 ± 0.443	High	[115]
*Bacillus subtilis*	PA-1	-	MIC: 0.0156 mg/mL	Moderate	[119]
*Enterobacter* *cloacae*	lasiocarpine	2 mg/mL	Zone of inhibition: 10.00 ± 0.41 mm	High	[120]
*Enterobacter* *cloacae*	lasiocarpine-*N*-oxide	2 mg/mL	Zone of inhibition: 9.00 ± 0.70 mm	High	[120]
*Escherichia coli*	lasiocarpine	2 mg/mL	Zone of inhibition: 12.00 ± 0.34 mm	High	[120]
*Escherichia coli*	lasiocarpine-*N*-oxide	2 mg/mL	Zone of inhibition: 9.00 ± 0.89 mm	High	[120]
*Escherichia coli*	clazamycin	-	MIC: 0.1 mg/mL	Low	[121]
*Proteus vulgaris*	PA-1	-	MIC: 0.0313 mg/mL	Moderate	[119]
*Pseudomonas* *aeruginosa*	PA-1	-	MIC: 0.0313 mg/mL	Moderate	[119]
*Pseudomonas* *aeruginosa*	clazamycin	-	MIC: 0.024–0.036 mg/mL	Low	[65]
*Staphylococcus* *aureus*	PA-1	-	MIC: 0.0039 mg/mL, 2*MIC within 8 h	Very High	[119]
*Staphylococcus* *epidermidis*	usaramine	1 mg/mL	Biofilm inhibition by 50%	Moderate	[122]
*Staphylococcus* *epidermidis*	PA-1	-	MIC: 0.0078 mg/mL	High	[119]
*Streptococcus pneumoniae*	heliotric acid	2 mg/mL	Zone of inhibition 13.64 ± 0.691 mm	High	[115]
*Streptococcus pneumoniae*	7-angeloyl heliotrine	2 mg/mL	Zone of inhibition 12.69 ± 0.317	High	[115]
Fungi					
*Aspergillus fumigatus*	heliotric acid	2 mg/mL	Zone of inhibition 10.59 ± 0.221	High	[115]
*Aspergillus niger*	PA-1	-	MIC: 0.125 mg/mL	Very low	[119]
*Candida albicans*	PA-1	-	MIC: 0.0625 mg/mL	Low	[119]
*Drechslera* *tetramera*	heliotridine	2 mg/mL	Zone of inhibition: 7.00 ± 0.51 mm	Moderate	[120]
*Fusarium* *moniliforme*	asiocarpine-*N*-oxide	2 mg/mL	Zone of inhibition: 7.00 ± 0.54 mm	High	[120]
*Penicillium chrysogenum*	7-angeloyl heliotrine	2 mg/mL	Zone of inhibition 11.61 ± 0.268	High	[115]
*Rhizoctonia phaseoli*	heliotric acid	2 mg/mL	Zone of inhibition 11.51 ± 0.187	High	[115]

**Table 9:** Potential applications of PAs.

PA	Potential Application	Refs.
Nervosin VII	human colorectal cancer cells	[106,107]
*N*-oxides of monocrotaline and heliotrine	a potential treatment for hepatomas– if significant hepatotoxicity targeted against tumor cells specifically	[112]
Indicine-*N*-oxide or its analogues	used as a microtubule-targeted anticancer drug	[111]
Retrorsine	antifungal agent against phytopathogenic fungi	[123]
Usaramine and monocrotaline	biomaterials surface coatings–usaramine antibiofilm activity and monocrotaline activity against *Trichomonas vaginalis*	[122]
PA-1	pro-drug against Gram-positive bacteria	[119]
Synthetic DHP	potential anticancer treatment–targets cancer cells specifically	[117]

## Supplementary Materials

The following supporting information can be downloaded at: https://www.mdpi.com/article/10.3390/ijms242316972/s1, refs. [9,27,34,39,112,124,125,126] are cited in supplementary materials.
