# Supplementary material for: Pyrrolizidine Alkaloids—Pros and Cons for Pharmaceutical and Medical Applications
Source: Int J Mol Sci. 2023 Nov 30;24(23):16972. doi: 10.3390/ijms242316972 (PMC10706944; doi:10.3390/ijms242316972)
Supplement: Supplementary file 1 [file ijms-24-16972-s001.zip › ijms-2721581-supplementary.pdf]

International Journal of
Molecular Sciences

MDPI

Review

Pyrrolizidine Alkaloids –Pros and Cons for Pharmaceutical and Medicinal Applications

Kavindi Jayawickreme[1,†], Dawid Świstak[1,†], Ewa Ozimek[2], Emilia Reszczyńska[3,4], Anna Rysiak[5], Anna Makuch-Kocka[6] and Agnieszka Hanaka[4*]

[1] Student Scientific Club of Phytochemists, Institute of Biological Sciences, Faculty of Biology and Biotechnology, Maria Curie-Skłodowska University, Akademicka St. 19, 20-033 Lublin, Poland
[2] Department of Industrial and Environmental Microbiology, Institute of Biological Sciences, Faculty of Biology and Biotechnology, Maria Curie-Skłodowska University, Akademicka St. 19, 20-033 Lublin, Poland
[3] Department of Biochemistry and Molecular Biology, Medical University of Lublin, Chodźki St. 1, 20-093 Lublin, Poland
[4] Department of Plant Physiology and Biophysics, Institute of Biological Sciences, Faculty of Biology and Biotechnology, Maria Curie-Skłodowska University, Akademicka St. 19, 20-033 Lublin, Poland
[5] Department of Botany, Mycology, and Ecology, Institute of Biological Sciences, Faculty of Biology and Biotechnology, Maria Curie-Skłodowska University, Akademicka St. 19, 20-033 Lublin, Poland
[6] Department of Pharmacology, Medical University of Lublin, Radziwiłłowska St. 11, 20-080, Lublin, Poland
* Correspondence: agnieszka.hanaka@mail.umcs.pl
† These authors contributed equally to this work.

Table S1. HPLC/UHPLC protocols of PAs detection. Abbreviations: HPLC/UHPLC - high-performance liquid chromatography/ ultra-high-performance liquid chromatography; PCX cartridge - a cation exchanger with mixed mode sorbent characteristics; GCB - graphitised carbon black; PSA - primary secondary amine; C18 - octadecylsilane.

Name of PAs	PAs-producing plants	Column	Extraction condition	Chromatography condition HPLC/UHPLC	Ref.
retrorsine;<br>senecivernine;<br>echimidine-*N*-oxide;<br>retrorsine-*N*-oxide;<br>sen-ecivernine-*N*-oxide;<br>concentration: > 2 µg/ml	*Atractylodis Macrocephalae Rhizoma*;<br>*Paeoniae Radix Alba;*<br>*Angelicae Sinensis Radix*;<br>*Bupleuri Radix*;<br>*Glycyrrhizae Radix* Et *Rhizoma* Praeparata Cum Melle	Thermo Hypersil Gold C18 (100 × 2.1 mm, 1.9 µm)	1% formic acid;<br>sonication: room temp., 20 min;<br>supernatant applied to the PCX cartridge;<br>pretreated: methanol + 0.05 M sulfuric acid solution (1:1, v/v);<br>rinsed: methanol + 0.05 M sulfuric acid solution;<br>PAs: washed with ammonia + methanol (1:3, v/v) solution	**mobile phase A:** 0.05% formic acid solution (containing 2.5 mM/L ammonium formate);<br>**mobile phase B:** methanol + 0.05% formic acid solution<br>(containing 2.5 mM/L ammonium formate);<br>**elution gradient:**<br>95% A, 0 min–0.5 min;<br>changed with a linear gradient to:<br>90% A, 0.5 min–5 min;<br>85% A, 5 min–11 min;<br>80% A, 11 min–13 min;<br>35% A, 13 min–14 min;<br>5% A, 14 min–16.5 min;<br>keeping isocratic elution for 2.5 min;<br>95% A, 16.5 min–16.6 min, 95% A, 16.6 min–18 min;<br>**flow rate:** 0.4 mL/min,<br>**column temperature:** 40°C,<br>**injection volume:** 2 µL in 50% methanol	[1]
green tea, black tea, herbal infusion	*Asteraceae: Eupatorium can-nabinum, Petasites hybridus, Senecio vulgaris, Tussilago farfara);*	Omega Polar C18 (2.1 × 100 mm, 1.6 µm;	aqueous extracts aliquot of the aqueous solution (10 ml) brought to a concentration of 1	**mobile phase A:** H_2O and<br>**mobile phase B:** MeCN<br>- both containing 0.1% of formic acid;	

lasiocarpine; retrosine; senecionine; monocrotaline; senkirkine; e.g., ~100 μg kg^{-1}	*Boraginaceae: Anchusa officinalis; Borago officinalis, Echium italicum, Heliotropium europaeum, Lithospermum officinale, Symphytum officinale*	Phenomenex) column	M of $MgSO_4{\cdot}7H_2O$, 1.5 M Na_2SO_4, pH 9.6; centrifugated: 5 min (13,000 rpm); 2 mL of the aqueous solution extracted with 2 mL of MeCN by vortexing for 1 min; centrifuged: 5 min (13,000 rpm) to achieve the phase separation; upper organic phase (MeCN)-dry under a gentle nitrogen flow; re-dissolved in 200 μL of H_2O/MeOH (7:3 v/v) for herbal infusions and tea	**elution gradient:** 2% B, 0–1 min; 2–8% B, 1–5.5 min; 8% B, 5.5–7.5 min; 8–12% B, 7.5–9.5 min; 12–18% B, 9.5–11 min; 18–20% B, 11–13 min; 20–40% B, 13–15 min; 40–60% B, 15–17 min; 60–80% B, 17–19 min; after each injection: washing (98% B, 4 min) and re-equilibration of the column (2% B, 5 min); **flow rate:** 0.4 mL/min, **column temperature:** 40 °C; **injection volume:** 5 μL in H_2O/MeOH (7:3, v/v)	[2]
heliotrine; europine	*Heliotropium dasycarpum* Ledeb.	Luna Omega Polar C18 column (3 μm, 250 x 4.6 mm)	dried plant material (3 kg); extraction: distilled MeOH twice (5 L, room temp.); solvent: evaporated under reduced pressure to get 75 g mass; methanolic extract (75 g) suspended in distilled water (500 mL); extracted with hexane and ethyl acetate; achieved fractions: hexane (35 g), ethyl acetate (22 g), and residual water (16 g)	**mobile phase A:** 0.1% formic acid in H_2O; **mobile phase B:** acetonitrile gradient: 10–100% B, 45 min; **flow rate:** 0.5 mL/min; **injection volume:** 3 μL; UV detector: 190–800 nm	[3]
heliotrine, heliotrine-*N*-oxide, retrorsine,	fresh tea leaves	HSS T3 column (2.1 × 100 mm,	leaves (1g); 10 mL of 0.1 M sulfuric acid solution; vortexed: 1 min;	**mobile phase A:** 0.1% formic acid + 1 mM ammonium formate in methanol'	

retrorsine-*N*-oxide,<br>denecionine,<br>senecionine-*N*-oxide,<br>jacobine,<br>jacobine-*N*-oxide,<br>intermedine,<br>intermedine-*N*-oxide,<br>seneciphylline,<br>seneciphylline *N*-oxide,<br>europine,<br>europine-*N*-oxide,<br>senkirkine	1.8 µm,<br>Waters)	ultrasonically extracted: 15 min;<br>centrifuged: 10 min (9,390g);<br>after repetition of extraction supernatant was combined;<br>2 mL aliquot of the supernatant placed with the adsorbents GCB:PSA:C18 (10:20:15 mg);<br>vortexed: 1 min;<br>centrifuged: 8 min (9,390g) at room temp.	**mobile phase B:** 0.1% formic acid + 1 mM ammonium formate in H_2O;<br>**elution gradient:**<br>10% A, 0–0.25 min;<br>10–30% A, 0.25–6.0 min;<br>30–40% A, 6.0–9.0 min;<br>40–98% A, 9.0–9.01 min;<br>held for 1.9 min;<br>98–100% A 11.0–11.1 min;<br>held for 2.9 min;<br>**flow rate:** 0.25 mL/min;<br>**column temperature:** 40 °C;<br>**injection volume:** 3 µL;	[4]

Table S2. Overview of PA-rich family *Boraginaceae*, where: life forms per – perennial plant, ann – annual, bienn – biennial. Geographical range and life forms based on the Plants of the World online [6] herbal raw materials according to Smith and Culvenor [7], and names of plant taxa according to the International Plant Names Index [8].

Genus	Species	Geographical range		Life form	Raw material
		Native	Secondary		
Alkanna	*orientalis*	S Eupope, W Asia		per	herba
	tinctoria	S Europe		per	herba
Amblynotus	*rupestris*	Asia C		subshrub	herba
Amsinckia	*carinata*	America N		ann	herba
	douglasiana	America N		ann	herba
	eastwoodiae	America N		ann	herba
	grandiflora	America N		ann	herba
	caycina	W, S America S		ann	herba
	lunaris	America N		ann	herba
	lycopsoides	America N		ann	herba
	lycopsoides × menziesii var. intermedia	America N	Europe, Asia	ann	herba
	menziesii	America N		ann	herba
	menziesii var. intermedia	S America N		ann	herba
	spectabilis var. microcarpa	America N		ann	herba
	specabtilis var. spectabilis	America N		ann	herba
	tessellata	America N, S		ann	herba
	tessellata var. gloriosa	America N		ann	herba
	tessellata var. tessellata	America N, S		ann	herba
	vernicosa var. furcata	America N		ann	herba
Anchusa	*arvensis*	Europe	America N	ann	herba
	hispida	Africa, Asia		ann	herba
	milleri	Africa, Asia		ann	herba
	officinalis	Europe, Asia		per	herba
	strigosa	Europe, Asia		per	herba
Arnebia	*decumbens*	Europe, Asia		ann	herba
	euchroma	Asia		per	herba
	hispidissima	Africa, Asia		ann	herba
Asperugo	*procumbens*	Europe, Asia	America N	ann	herba
Borago	*officinalis*	Europe	America N	ann	herba, seeds
Caccinia	*macranthera var. crassifolia*	Asia		per	herba
Cerinthe	*glabra*	Asia		bienn	herba
	minor	Europe, Asia		ann	herba
Cordia	*myxa*	Asia		per/tree	herba
	sinensis	Africa, Asia		per/shrub/tree	herba
Cryptantha	*cana*	America N, S		ann	herba

	clevelandii	America N, S		ann	herba
	confertiflora	America N, S		per	herba
	crassipes	America N, S		per	herba
	fendleri	America N, S		per	herba
	flava	America N, S		per	herba
	inequata	America N, S		per	herba
	jamesii	America N, S		per	herba
	leiocarpa	America N, S		per	herba
	thyrsiflora	America N, S		per	herba
	utahensis	America N, S		per	herba
	virgata	America N, S		per	herba
	virginiensis	America N, S		per	herba
Cynoglossum	*amabile*	Asia	America N, S	ann/bienn	herba
	australe	Australia	America N, S	per	herba
	clandestinum	Europe	America N, S	bienn	herba
	columnae	Europe	America N, S	ann	herba
	creticum	Europe	America N, S	per	root, herba
	furcatum	Asia	America N, S	per	herba
	germanicum	Europe, Asia	America N, S	bienn	herba
	glochidiatum	Europe, Asia	America N, S	per	herba
	lanceolatum	Africa, Asia	America N, S	per	herba
	latifolium	Asia	America N, S	per	herba
	macrostylum	Europe	America N, S	per	herba
	montanum	Europe, Asia	America N, S	per	herba
	nervosum	Europe, Asia	America N, S	per	herba
	officinale	Europe	America N, S	per	root, herba
	pictum	Europe	America N, S	per	root, herba
	viridiforum	Asia	America N, S	per	root, herba
Echium	*amoenum*	Europe, Asia		bienn	herba
	angustifolium	Europe		per	herba
	diffusum	Europe		per	herba
	glomeratum	Europe, Asia		bienn	herba
	horridum	Africa		ann/per	herba
	humile	Africa, Europe		per	herba
	hypertropicum	Africa		shrub	herba
	italicum	Europe, Asia		bienn	herba
	plantagineum	Europe		ann/bienn	herba
	pininana	Europe		bienn/subshrub	herba
	rauwolfii	Africa, Asia		ann	herba
	angustfoilum ssp. sericeum	Africa, Asia		per	herba

	simplex	Europe		bienn/subshrub	herba
	stenosiphon ssp. stenosiphon	Africa		subshrub/shrub	herba
	tuberculatum	Africa, Spain		bienn	herba
	vulgare	Europe, Asia		ann/bienn	herba
	wildpretti	Europe		bienn/subshrub	herba
Ehretia	*aspera*	Asia		shrub/tree	herba
Euploca	*bracteatum*	Australia		ann	herba
	bursiferum	America C		ann	herba
	ovalifolium	N Africa, Asia		ann/subshrub	herba
	parviantrum	C, E Australia		ann	herba
	procumbens	S America N		ann	herba
	queretaroanum	S America N		ann	herba
	racemosum	S America N		subshrub	herba
	marifolia ssp. marifolia	S Asia		ann/subshrub	herba
	sessei	S America N		ann	herba
	humilis	S America N		subshrub	herba
	wigginsii	S America N		ann	herba
Hackelia	*californica*	America N		per	herba
	uncinata	C Asia		per	herba
	floribunda	America N		bienn/per	herba
	velutina	America N		per	herba
Heliotropium	*acutiflorum*	Asia		subshrub	herba
	amplexicaule	America S		subshrub	herba
	angiospermum	America S, C, N		ann/subshrub	herba
	arbainense	Africa, Asia		subshrub	herba
	arborescens	Anerica S		subshrub/shrub	herba
	arguzioides	Europe, Asia		subshrub	herba
	bacciferum	Africa		subshrub	herba
	bovei	Europe		ann	herba
	arboreum	E Africa, W Australia		shrub	herba
	sibiricum	SE Europe, Asia		per	herba
	circinatum	Asia		ann	herba
	confertifolium	Asia		ann	herba
	crassifolium	Asia		ann	herba
	curassavicum	Australia, America N, S	Africa N	ann/subshrub	herba, seeds
	curassavicum var. argentinum	America S, Australia	Africa N	ann/subshrub	herba

	curassavicum var. curassavicum	Australia, America N, S	Africa N	ann/subshrub	herba
	dasycarpum	Asia		subshrub	root, herba
	digynum	Africa N, Asia		subshrub	herba
	disciforme	Asia		per	herba
	dissitiflorum	Asia		per	herba
	elipticum	Asia		per	herba, seeds
	esfandiarii	Asia		per	herba
	europaeum	Europe, Asia		per	herba
	floridum	America S		subshrub	herba
	foliosisimum	America N		subshrub	herba
	fruticosum	America N, S		per	herba
	hirsutissimum	Europe		per	herba
	indicum	America S	America C, Africa C, Asia SE	per	herba
	keralense	Asia		per	herba
	lasiocarpum	Africa, Asia		per	herba
	marifolium	Asia		per	herba
	maris mortui	Asia		per	herba
	megalanthum	America S		subshrub/shrub	herba
	molle	S America N		ann	herba
	olgae	C Asia		per	root, herba
	popovii ssp. gillianum	Asia		per	seed
	ramosissimum	NE Africa, W Asia		subshrub	herba
	rotundifolium	N Africa		subshrub	herba
	curassavicum var. obovatum	N America N		per	herba
	steudneri	S, N Africa		subshrub/shrub	herba
	ophiglosum	NE Africa, W Asia		subshrub	herba
	suaveolens	SE, E Europe, W Asia		ann	herba
	zeylandicum	S Africa, S Asia		per	herba
	sarmentosum	E Asia, Australia		shrub	herba
	supinum	E, S, C Europe, Africa, W Asia		ann	leaf, root, herba
Euploca	*humilis*	S America N, S America S		subshrub	herba
	transalpinum	S America N, S America S		subshrub/shrub	herba

	transalpinum var. transalpinum	America N, C		subshrub/shrub	herba
	dasycarpum ssp. transoxanum	C Asia		subshrub	herba
	intermedia	C, E Asia		ann	herba
	squarrosa	Eurasia	America N	bienn	herba
	spinocarpos	N Africa, W Asia		ann	herba
Lindelofia	*anchusoides*	C Asia		per	herba
	stylosa ssp. stylosa	C Asia		per	herba
	longiflora	C Asia		per	herba
	olgae	C Asia		per	herba
	stylosa ssp. pterocarpa	C Asia		per	herba
	longiflora var. longiflora	C Asia		per	herba
	stylosa	C Asia		per	seed
	tschimganica	C Asia		per	herba
Lithospermum	*canesens*	C America N		per	herba
	erythrorhizon	E Asia		per	herba
	officinale	Eurasia	E America N	per	herba
Lithodora	*fruticosa*	N Africa, France, Spain		subshrub	herba
Moltikiopsis	*ciliata*	N Africa, W Asia		subshrub	herba
Myosotis	*scorpioides*	Eurasia	America N	per	herba
	sylvatica	Eurasia	America N	per	herba
Neatostema	*apulum*	S Europe, N Africa, W Asia		ann	herba
Nonea	*lutea*	W Asia, C Europe		ann	herba
	setosa	C Asia		per	herba
	echioides	S Europe, W Asia		ann	herba
Omphalodes	*verna*	S Europe	C, N Europe, E America N	per	herba
Onosma	*alborosea*	W Asia		subshrub	herba
	arenaria	C, SE Europe		bienn/per	herba
	arenaria ssp. pennina	S Europe		per	herba
	erecta	S Europe		per/subshrub	herba
	hetrophyllum	S Europe		per/subshrub	herba
	leptantha	S Europe		per	herba
	stellulata	S Europe		per	herba
Paracaryum	*himalayense*	C Asia		ann	herba
Microparacaryum	*intermedium*	ES Europe, W Asia		ann	herba
	regulosum	ES Europe, W Asia		bienn/per	herba
Cynoglossum	*zeylandicum*	S Asia		bienn	root, herba
Pulmonaria	*obscura*	Europe to W. Siberia		per	herba

Rindera	*austroechinata*	C Asia		per	leaf, root, herba, seed
	tetraspis	E Europe, W Asia		per	herba
	echinata	C Asia		per	herba
	oblongifolia	C Asia		per	herba
	umbellata	SE Europe		per	herba
Solenanthus	*circinnatus*	W, C Asia		per	herba, seed, root
	coronatus	S Europe		per	herba
	karateginus	C Asia		per	herba
	turkestanicus	W, C Asia		per	herba
Symphytum	*aintabicum*	W Asia		per	root, herba
	asperum	W Asia	America N, N Europe	per	root, herba
	bohemium	E. Europe to Caucasus.	S, W Europe	per	root, herba
	caucasium	Caucasus	S, W Europe	per	root, herba
	consolidum	Europe		per	root, herba
	grandiflorum	Europe	N, C America S; C Asia	per	root, herba
	ibericum	W Asia	W Europe	per	root, herba
	officinale	Eurasia	America N, C, S	per	root, herba
	orientale	SE, E. Europe, W Asia	C Europe	per	root, herba
	officinale ssp. officinale	Eurasia	America N	per	root, herba
	sylvaticum ssp. sepulcare var. sepulcare	W Asia		per	root, herba
	tanaiense	C, E Europe		per	root, herba
	tuberosum	C, W Europe		per	root, herba
	× uplandicum	C Asia	Europe	per	root, herba
Trahlenthus	*hissaricus*	Asia		per	herba
	korolkovii	Asia		per	herba
Trichodesma	*africanum*	Africa		per	herba
	ehrenbergii	Africa, Asia		ann	herba
	incanum	Asia		per	herba, seed, root
	zeylanicum	Africa, Asia		ann	herba
Ulugbekia	*tschimganica*	Asia		per	herba

Table S3. Dynamics of PAs occurrence in species of the family *Boraginaceae*. Group I – rare, exclusive compounds, found in 1 to 3 species; Group II – moderately frequent compounds, recognized in 4 to 9 species; Group III – frequent compounds, found in 10 to 15 species; Group IV – very frequent compounds, recognized in 16 to 20 species; Group V – common compounds, found in more than 20 species. In each group metabolites are ranked alphabetically by increasing occurrence in plants. List of the compounds was prepared according to El-Shazly and Wink [5]. Metabolites from each group ranked alphabetically by increasing occurrence in plants.

**Number and Name of PA**							
**Group I**							
1	3'-Acetylcanesine	45	Dihydroechinatine	89	(7S,8S) Petranine	133	Erythro-2'',3''-chloro-2''-hydroxyechiumine
2	3'-Acetylcanescenine	46	Echiupine	90	Pictumine	134	Heliospathuline
3	7-Acetyl-9-curassa-voylheliotridine	47	Echivulgarine	91	Platynecine	135	Heliotridine
4	7-Acetyl-9-(2,3-dihy-droxybutryl) ret-ronecine	48	Ehretinine	92	Platynecine N-oxide 2S-hy-droxy-2S (1S-hydroxyethyl) -4-methyl-pentanosyl ester	136	9-(3'-Isovaleryl)viridi-flory retronecine
5	7-Acetyl-9-(2-dimethyl-butryl) retronecine	49	7-Epi-echimiplatine	93	Punctanecine	137	Longitubine
6	7-Acetylechinatine	50	1α-2α-Epoxy-1β-hy-droxymethyl-8α-pyrrol-izidine	94	Pycnanthine	138	7-(2-Methylbutyryl)ret-ronecine
7	3'- Acetylechihumiline	51	Floridanine	95	Retronecine-7:9- dibenzoate	139	7-(2-Methylbutyryl)-9-(2,3-dihydroxy-butyryl)retronecine
8	3'-Acetylechiumine	52	Floridimine	96	Retronecine 2S-hydroxy-2S(1S-hydroxyethyl)-4-me-thyl-pentanosyl ester	140	Monocrotaline
9	3'-Acetylechimidine	53	Floridine	97	Retronecine 2S-hydroxy-2S(1R-hydroxyethyl)-4-me-thyl-pentanosyl ester	141	Neolatifoline
10	5'-Acetylechimidine	54	Floridinine	98	Scorpioidine	142	7-Senecioylhelotridine
11	5'-Acetyleuropine	55	Hackelidine	99	7-Senecioylrinderine	143	9-Senecioylretronecine
12	7-Acetyleuropine	56	Helibracteatine	100	7-Senecioyllycopsamine	144	7-Tigloyl-9-(2,3-dihy-droxypropanoyl)ret-ronecine
13	7-Acetyl-9-(2-hydroxy-3-methylbutryl) ret-ronecine	57	Helibractinecine	101	Sincamidine	145	7-Tigloyllycopsamine
14	5'-Acetyllasiocarpine	58	Helibracteatinecine	102	Senkirkine	146	Trachelanthine
15	7-Acetyl-9-latifolylret-ronecine	59	Helibracteatinine	103	Strigosine	147	Triangularine
16	3'-Acetyllithosenine	60	Heliofoline	104	Supinidine N-oxide 2S-hy-droxy-2S(1S-hydroxyethyl)-4-methyl-pentanoyl ester	148	Triangularicine
17	7-Acetyl-9-(2-methyl-butyryl) retronecine	61	Helindicine	105	Thesinine	149	Viridantine
18	7-Acetylretronecine	62	Heliocoromandaline	106	Thesinine-4'-O-β-D-gluco-side	150	3'-Acetylheliosupine
19	7-Acetyl-9-sarracinoyl retronecine	63	Heliocurassavine	107	7-Tigloyl-9-(2-deoxy-2-me-thyl) echimidinylheliotridin	151	9-Acetytessellatine
20	7-Acetylscorpioidine	64	Heliocurassavicine	108	7-Tigloylheliotridine	152	7-Angeloylheliotridine
21	3'-Acetylsupinine	65	Heliocurassavinine	109	7-Trachelanthyl-laburnine	153	Asperumine

22	3'-Acetyltessellatine	66	Heliospathine	110	7-Trachelanthylretronecine	154	Carategine
23	3'-Acetyltrachelan-thamine	67	Heliotridine 2S-hy-droxy-2S (1S-hydroxy-ethyl0-4-methyl-penta-noyl ester	111	Transalpinecine	155	Curassavine
24	9-Acetyltrachelantham-ine	68	Heliovicine	112	Uluganine	156	Cynaustrine
25	3'-Acetylviridiflorine	69	Heliscabine	113	Vulgarine	157	3',7-Diacetylinterme-dine
26	7-Acetylvulgarine	70	Hydroxymyoscorpine	114	3'-Acetylfurcatine	158	thero-2'',3''-Dihydroxy-echiumine
27	7α-Angeloyl-1-chloro-methy-1,2-dihydro-pyrrolizidine	71	Ilamine	115	3'-Acetylindicine	159	Echimiplatine
28	7-Angeloyl-9-(2,3-dihy-droxybutyryl) heliotri-dine	72	Indicinine	116	3'-Acetylmyscorpine	160	Echiuplatine
29	7-Angeloyl-1-formyl-6,7-dihydro-5H-pyrrol-izidine	73	Isoechinatine	117	3'-Acetylrinderine	161	Furcatine
30	7-Angeloylechinatine	74	Isolycopsamine	118	9-(3'-Acetyl)viridiflory ret-ronecine	162	Incanine
31	7-Angeloylrinderine	75	Isoretronocanol (or its isomer)	119	9-(3'-Acetylviridifloryl) turniforcidine	163	7-Senecioylretronecine
32	9-Angeloyltrachelan-thamidine	76	Lactodine	120	Anadoline	164	Symlandine
33	7-Angeloyl-9-(+)-tra-chelanthylheliotridine	77	9-Latifolylretronecine	121	7-Angeloyl-9-(2,3-dihydrox-ypropanoyl)retronecine	165	Symviridine
34	9-(Butyryl-2-ene) su-pinidine	78	Lindelofamine	122	7-Angeloyl-9-(hydroxypro-penoyl) retronecine	166	Trichodesmine
35	Canescine	79	Lithosenine	123	7-Angeloyllycopsamine	167	Turkestanine
36	Canescenine	80	Macrophylline	124	7-Angeloyl-9-(2-methyl-butyryl)heliotridine	168	Uplandicine
37	Cryptanthine	81	Macrotamine	125	7-Angeloyl-9-(2-methyl-butyryl)retronecine		
38	Curassanecine	82	Megalanthonine	126	Coromandaline		
39	9-Curassavorylheliotri-dine	83	Methyechiuplatine	127	Coromandalinine		
40	Cynoglossamine	84	1-Methylene-8α-pyrrol-izidine	128	Curassavinine		
41	Dehydroheliotrine	85	9-(2-Methylbutyryl) ret-ronecine	129	3',7-Diacetyllycopsamine		
42	5-Deoxylasiocarpine	86	Neocoromandaline	130	Dihydroxytriangularine		
43	3',9-Diacetyltessellatine	87	Onosmerectine	131	Dihydroxytriangularicine		
44	5,6-Dihydro-7,9-di-methoxy-7H-pyrroliz-ine	88	(7S,8R) Petranine	132	2'',3''-Epoxyechiumine		

**Number and Name of PAs**							
	**Group II**		**Group III**		**Group IV**		**Group V**
1	3'-Acetylechinatine	1	7-Acetyllycopsamine	1	3'-Acetylintermedine	1	Retronecine

2	7-Angeloyl-9-(2,3-dihydroxybutyryl)retronecine	2	Echiumine	2	Lasiocarpine	2	7-Acetyllycopsamine
3	Echihumiline	3	Trachelanthamine	3	7-Angeloylretronecine	3	Heliotrine
4	7-(2-Methylbutyryl)-9-echimidinyl retronecine	4	Symphytine	4	7-Acetylintermedine	4	Echimidine
5	7-Tigloyl-9-(2,3-dihydroxybutyryl) retronecine	5	3'-Acetyllycopsamine	5	Amabiline	5	Supinine
6	7-Tigloyl-9-(2-methybutyryl)retronecine	6	9-Angeloylretronecine			6	Echinatine
7	9-Angeloyl-7-viridiflorylretronecine	7	Europine			7	Intermedine
8	Cynaustraline	8	Heliosupine			8	Lycopsamine
9	Cynaustraline	9	Rinderine				
10	Echimidine isomer (tigloyl)	10	Supinidine				
11	Leptanthine	11	Viridiflorine				
15	Subulacine	12	Lindelofidine				
13	7-Tigloylretronecine	13	Tessellatine				
14	7-Viridiflorylretronecine	14	Trachelanthamidine				
15	Heleurine						
16	Indicine						
17	Latifoline						
18	Lindelofine						
19	Myoscorpine						
20	9-Tigloylretronecine						
21	7-Angeloylheliotridine						

References

1. Cheng, S.; Sun, W.; Zhao, X.; Wang, P.; Zhang, W.; Zhang, S.; Chang, X.; Ye, Z. Simultaneous determination of 32 pyrrolizidine alkaloids in two traditional Chinese medicine preparations by UPLC-MS/MS. *J. Anal. Methods Chem.* **2022**, *2022*, doi:10.1155/2022/7611501.

2. Rizzo, S.; Celano, R.; Piccinelli, A.L.; Serio, S.; Russo, M.; Rastrelli, L. An analytical platform for the screening and identification of pyrrolizidine alkaloids in food matrices with high risk of contamination. *Food Chem.* **2023**, *406*, doi:10.1016/j.foodchem.2022.135058.

3. Mukhtar, M.; Saleem, M.; Nazir, M.; Riaz, N.; Shafiq, N.; Saleem, H.; Tauseef, S.; Khan, S.; Ehsan Mazhar, M.; Bakhsh Tareen, R.; et al. Identification of pyrrolizidine alkaloids and flavonoid glycosides through HR-LCMS/MS analysis, biological screening, DFT and molecular docking studies on Heliotropium dasycarpum Ledeb. *Arab. J. Chem.* **2023**, *16*, doi:10.1016/j.arabjc.2023.104655.

4. Jiao, W.; Zhu, L.; Shen, T.; Wang, L.; Li, Q.X.; Wang, C.; Wu, X.; Chen, H.; Hua, R. Simultaneous determination of 15 pyrrolizidine alkaloids and their N-oxides in weeds, soil, fresh tea leaves, and tea: exploring the pollution source of pyrrolizidine alkaloids in tea. *Food Chem.* **2024**, *434*, 137305, doi:10.1016/j.foodchem.2023.137305.
5. El-Shazly, A.; Wink, M. Diversity of pyrrolizidine alkaloids in the Boraginaceae structures, distribution, and biological properties. *Diversity* **2014**, *6*, 188–282, doi:10.3390/d6020188.
6. Plants of the World online; Board of Trustees of the Royal Botanic Gardens, Kew Available online: https://powo.science.kew.org.
7. Culvenor, C.C.J. Tumor-Inhibitory activity of pyrrolizidine alkaloids. *J. Pharm. Sci* **1968**, *57*, 1112–1117.
8. IPNI – The International Plant Names Index Available online: https://www.ipni.org.
